# A physico-mechanical model of postnatal craniofacial growth in human

**DOI:** 10.1016/j.isci.2024.110617

**Published:** 2024-07-29

**Authors:** Ce Liang, Arsalan Marghoub, Antonio Profico, Costantino Buzi, Marius Didziokas, Lara van de Lande, Roman Hossein Khonsari, David Johnson, Paul O’Higgins, Mehran Moazen

**Affiliations:** 1 Department of Mechanical Engineering, University College London, London WC1E 7JE, UK; 2 Department of Biology, University of Pisa, 56126 Pisa, Italy; 3 Institut Català de Paleoecologia Humana i Evolució Social (IPHES-CERCA), 43007 Tarragona, Spain; 4 Departament d’Història i Història de l’Art, Universitat Rovira i Virgili, 43002 Tarragona, Spain; 5 Department of Oral and Maxillofacial Surgery, Erasmus Medical Centre, 3015 GD Rotterdam, the Netherlands; 6 Craniofacial Growth and Form Laboratory, Hôpital Necker–Enfants Malades, Assistance Publique - Hôpitaux de Paris, Faculté de Médecine, Université Paris Cité, 75015 Paris, France; 7 Oxford Craniofacial Unit, Oxford University Hospital, Oxford OX3 9DU, UK; 8 Department of Archaeology and Hull York Medical School, University of York, York YO10 5DD, UK

**Keywords:** Developmental biology, In silico biology, Biological constraints

## Abstract

Our fundamental understanding of the physico-mechanical forces that drive the size and shape changes of the cranium during ontogeny are limited. Biomechanical models based on finite element method present a huge opportunity to address this critical gap in our knowledge. Here, we describe a validated computational framework to predict normal craniofacial growth. Our results demonstrated that this approach is capable of predicting the growth of calvaria, face, and skull base. We highlighted the crucial role of skull base in antero-posterior growth of the face and also demonstrated the contribution of the maxillary expansion to the dorsoventral growth of the face and its interplay with the orbits. These findings highlight the importance of physical interactions of different components of the craniofacial system. The computational framework described here serves as a powerful tool to study fundamental questions in developmental biology and to advance treatment of conditions affecting the craniofacial system such as craniosynostosis.

## Highlights

• Presented a geometrically complete biomechanical newborn skull model
• Developed a computational framework to predict postnatal craniofacial growth in human
• Investigated the mechanical interactions among calvaria, skull base, and face
• Evaluated the level of mechanical strain that craniofacial system experiences

## Graphical abstract

**Figure:**
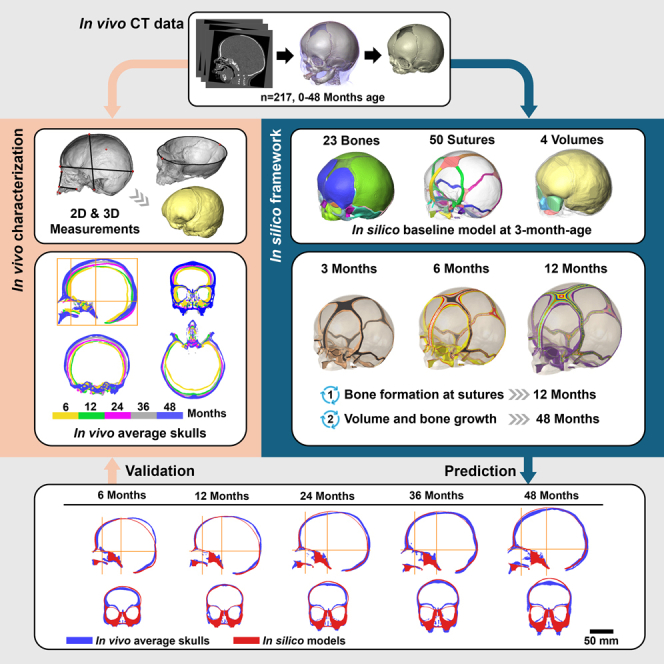

## Introduction

The human skull can be divided into the calvaria, base, and face. Each consists of several bony elements, connected via fibrous or cartilaginous joints, housing key organs such as the brain, eyes, and tongue, and provide support for the muscles of mastication and neck.^1^^,^^2^^,^^3^^,^^4^^,^^5^ During the early stages of postnatal ontogeny, internal organs increase in volume hand in hand with their overlying complex of bones, joints, and soft tissues. Calvarial joints (sutures) accommodate rapid brain growth,^4^^,^^6^^,^^7^ skull base joints (synchondroses) contribute to cranial and facial growth,^8^ and facial sutures facilitate the expansion of the orbits, intraoral, and nasal cavities.^9^ All joints undergo tissue differentiation in response to various chemical and physical signals and sutural spaces reduce gradually to micro- or nanometer gaps.^10^^,^^11^^,^^12^^,^^13^ At the same time, bones gradually thicken and adapt to withstand the forces arising from mastication and posture.^14^^,^^15^^,^^16^ By mid-childhood (6–7 years of age), the cranium becomes a rather more solid structure, protecting the brain, and other internal organs and has achieved about 90% of adult size. It continues to grow and develop at a slower rate until adulthood (∼25 years of age).^17^

Regulation and control of postnatal craniofacial ontogeny at the macro-level are critical in investigating the mechanobiology of the natural processes of craniofacial system development in the first few years of life. However, what drives changes in the form of cranial components and the interactions among components is debated.^18^ The functional matrix theory^19^^,^^20^ posits that the growth of skeletal elements is secondary to that of the soft tissues which regulate skeletal morphogenesis through their expansion (e.g., growth of the neurocranium is driven by brain growth) or action (periosteal matrices driven by muscle activity), with strong interactions between these functional matrices due to their anatomical proximity (for instance the anterior skull base corresponds to the orbital roofs). Alternatively, primary growth center theory^21^ posits that cartilages of the nasal region and condyles drive vertical and anteroposterior growth of the facial skeleton. More precisely, the dynamic developmental spatial and mechanical interactions among developing parts of the cranium and soft tissues clearly play a role in maintaining normal growth, because perturbation of one part has diffused consequences in the whole cranium, as in the case of craniosynostosis.^22^ Yet we barely understand the nature, strength, and time course of these interactions^5^^,^^15^^,^^23^ during normal growth. In two dimensions (2D), theories on global craniofacial growth have led to the development of widely used clinical tools such as Delaire’s cephalometric analysis,^24^ but these approaches require both scientific validation and extension to three dimensions (3D).^25^ Understanding of the growth processes is naturally crucial to advancing treatment of pathological conditions, and one way of assessing current hypotheses, concerning the role of dynamic developmental spatial and mechanical interactions in craniofacial growth and development, is to model them computationally.

**Figure 1:**
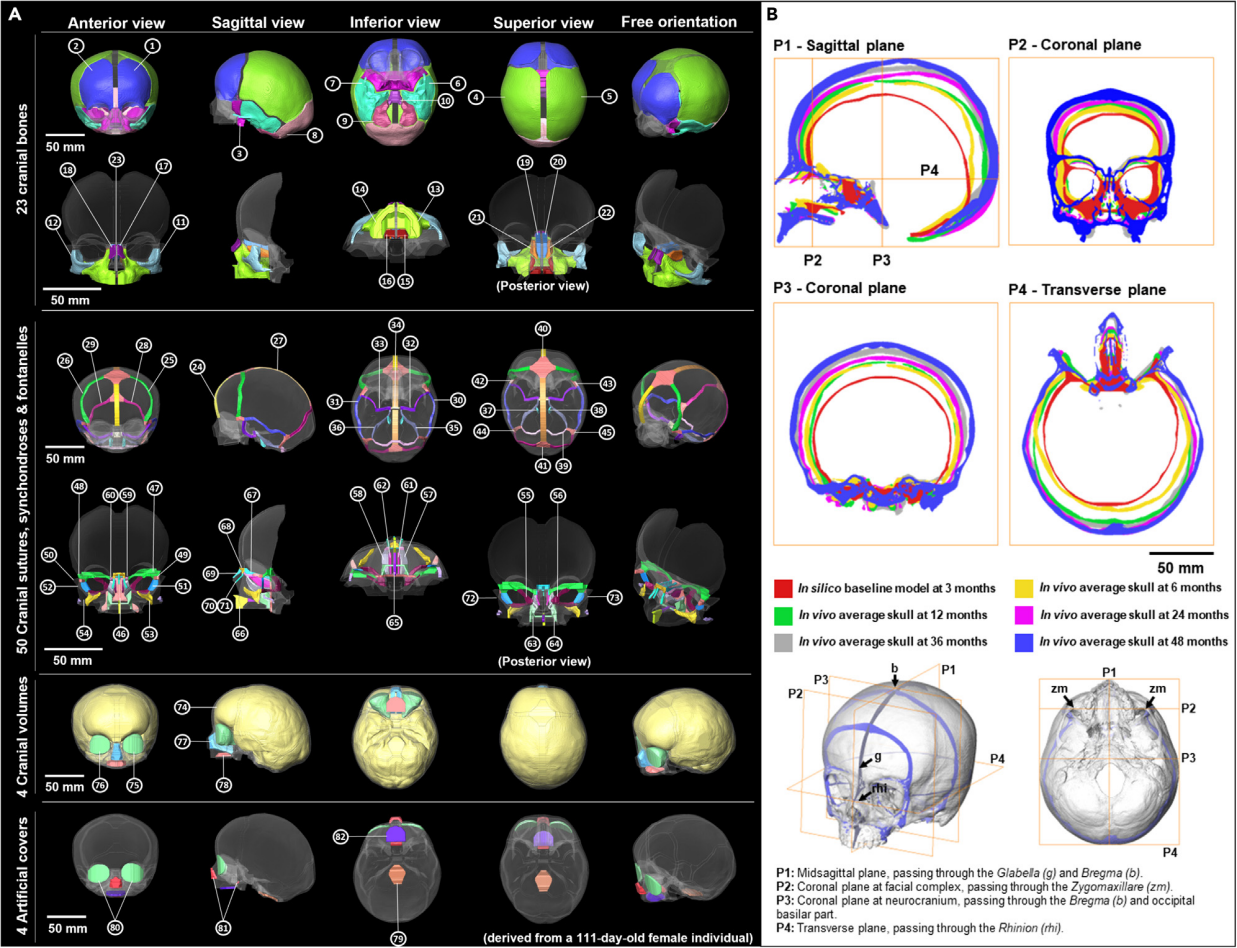
*In silico* model and *in vivo* average skulls at 3, 6, 12, 24, 36, and 48 months of age For a Figure360 author presentation of this figure, see https://doi.org/10.1016/j.isci.2024.110617. (A) *In silico* baseline model derived from a 111-day-old female individual segmented with 23 cranial bones (no. 1–23), 40 cranial sutures (no. 24–33, 35, 46–73), 4 synchondroses (no. 34, 37–39), 6 fontanelles (no. 40–45), 4 cranial volumes (inc. paired orbital volumes, no. 74–78), and 4 artificial covers (no. 79–82). More details of *in silico* model are shown in Table S1. (B) Cross-sections of pre-aligned *in silico* baseline model and *in vivo* average skulls in four standard anatomical planes.

**Figure 2:**
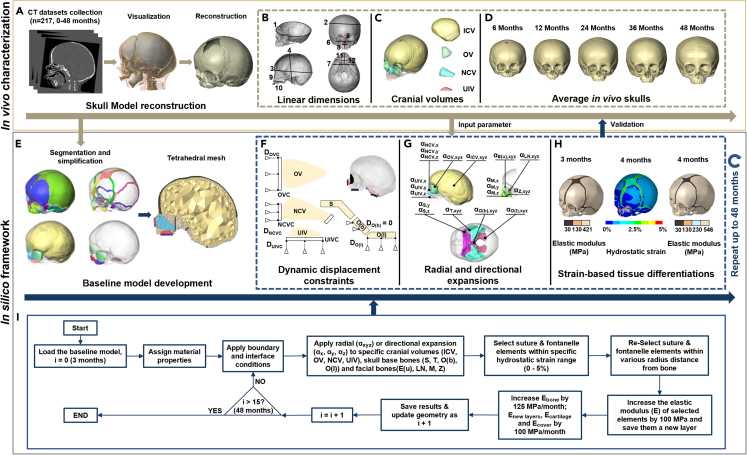
Overview of *in vivo* characterization and *in silico* simulation Skull models were reconstructed from clinical CTs of 217 normal individuals from birth to 4 years of age (A), a normative dataset including 12 linear cranial dimensions (B) and 4 cranial volumes (C) was created, then 5 average skulls (D) were selected as the references for simulation. A 3D finite-element baseline model was developed based on a 111-day-age female individual (E). After assigning the initial material properties, boundary and interface conditions for the current age were applied (F). Cranial volumes (intracranial volume-ICV, orbital volume-OV, nasal cavity volume-NCV and upper intraoral volume-UIV), skull base bones (sphenoid-S, temporal bone-T, basilar and lateral portions of occipital bone-O(b) and O(l)), and midfacial bones (upper portion of ethmoid bone-E(u), lateral wall of nasal cavity-LN, zygoma-Z and maxilla-M) were expanded radially or directionally to the target volumes of the next age (G). An algorithm of strain-based tissue differentiation with suture-specific closure rates was applied to replicate bone formation at sutures and stiffness differentiations (H). These processes (F–H) were repeated until 48 months of age. The flow diagram shows the whole computational framework (I).

**Figure 3:**
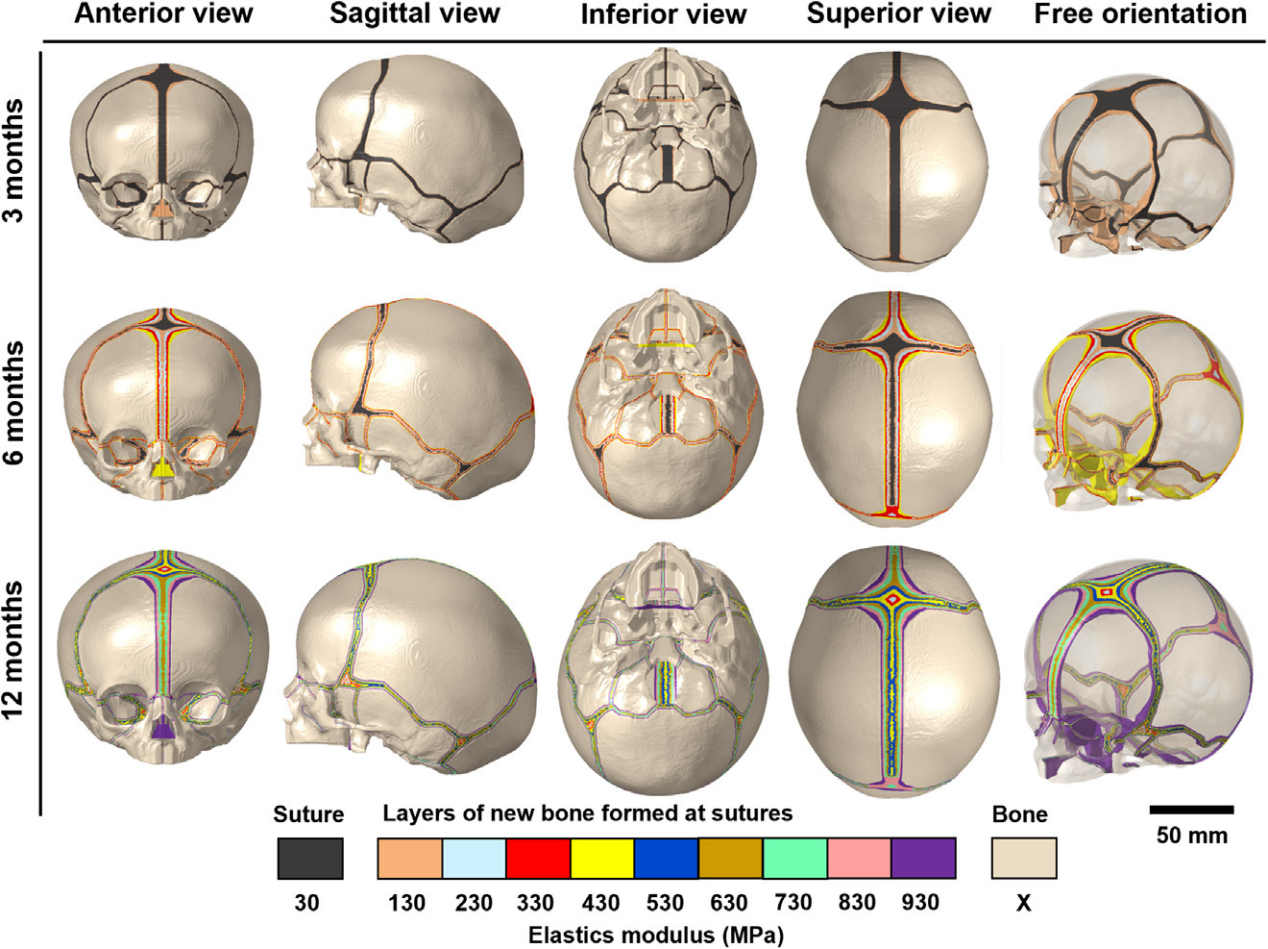
*In silico* images of strain-based tissue differentiation at sutures (including fontanelles and synchondroses) applied with suture-specific closure rates from 3 to 12 months of age See Table 1 for the details of various closure rates. Note that, bone formation at sutures and stiffness differentiations were both simulated up to 12 months of age, i.e., the elastic modulus of bone components (X) is increased by 125 MPa/month, from 421 MPa at 3 months to 1546 MPa at 12 months. No new layer is formed at sutures and only a stiffness differentiation algorithm was applied after 12 months, i.e., the elastic modulus of these 9 newly formed layers and bone components (X) are increased by 100 MPa/month and 125 MPa/month respectively up to 48 months.

**Figure 4:**
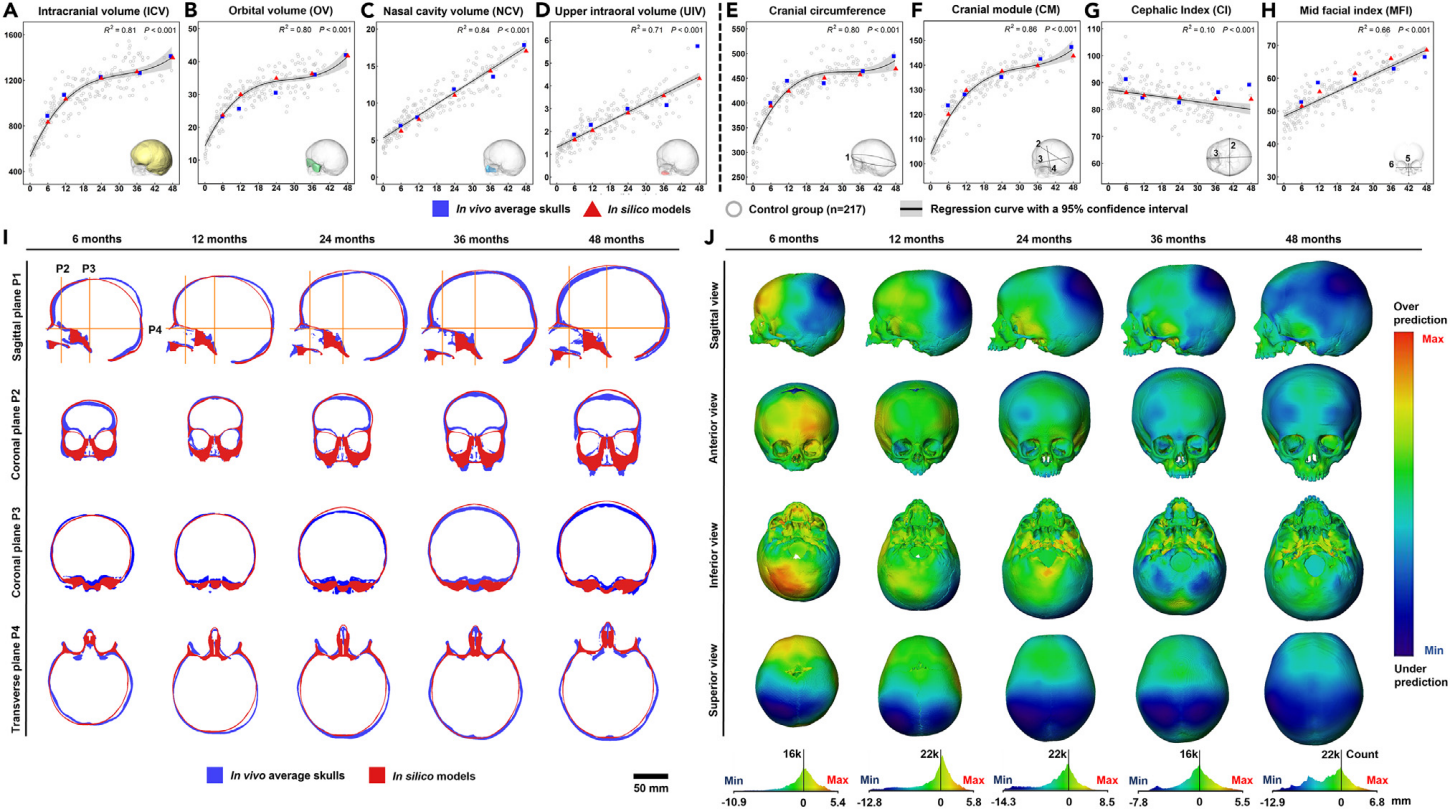
*In silico* models versus *in vivo* average skulls at 6, 12, 24, 36, and 48 months of age (A–D) scatterplots of the comparisons between the predicted volumes (ICV, OV, NCV, and UIV) of *in silico* models and measured values of average skulls at each age. (E–H) scatterplots of the comparisons in the measurements of cranial circumference, cranial module (CM), cephalic index (CI), and mid-facial index (MFI) between *in silico* models and average skulls at each age. Note that the corresponding data from the control group of 217 normal individuals were also plotted in the scatterplots, indicating the overall changes in and distributions of the normative dataset. The significance level (*p* value) and coefficient of determination (R^2^) were reported along with each regression in (A–H). (I) 2D morphological comparisons between pre-aligned *in silico* models and average skulls at each age visualized by plotting cross-sections in four planes. (J) 3D morphological comparison of overall shape between the external surface of *in silico* models and average skulls through distance plots. Each skull was scaled individually with the maximum and minimum values, and the color chart mapped in the histogram displayed below each age shows the distribution of shape differences across the skull.

**Figure 5:**
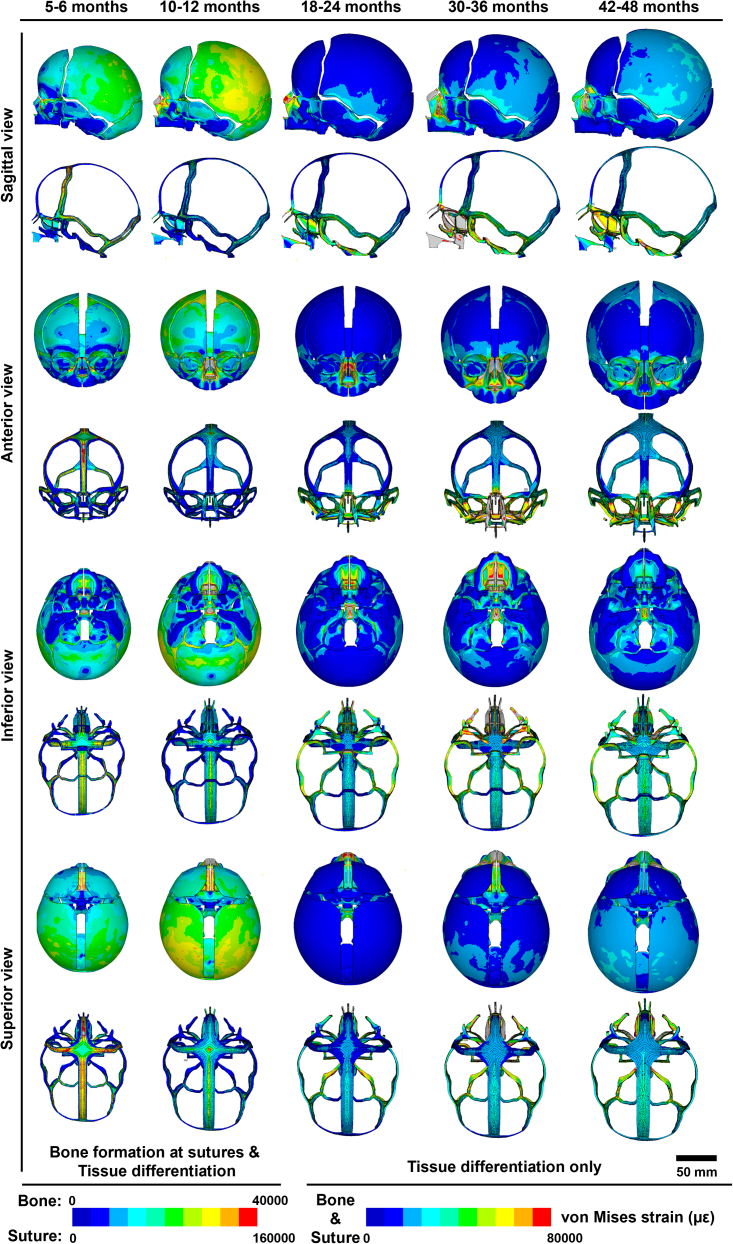
Changes in strain pattern across the skeleton and craniofacial sutures at 6, 12, 24, 36, and 48 months of age during growth von Mises strains (με) were derived from one load-step between former age to the target age and mapped on the *in silico* model at target age. Skeletal and sutural components were scaled separately using different ranges of strain before 12 months to represent both bone formation at sutures and tissue differentiation, between 12 and 48 months the same strain range was used for the skeletal and sutural components. Note that the results of the first principal strain are attached in Figure S12.

In a series of studies, we previously developed a computational framework based on finite-element (FE) method to predict calvarial growth.^26^^,^^27^^,^^28^^,^^29^^,^^30^^,^^31^^,^^32^ This framework simulates capsular matrix expansion using changes in intracranial volume (brain) as the main driver of calvarial growth, integrating contact mechanics, and strain-based tissue differentiation at the calvarial sutures. We tested and validated that framework to predict calvarial growth in newborn mice (i.e., wild-type and craniosynostotic mice),^27^^,^^28^ human infants^26^ and demonstrated the application of such modeling in optimizing management of sagittal craniosynostosis, a condition affecting growth of the calvaria.^29^^,^^30^^,^^31^^,^^32^ To date, the main limitation of this framework is that it does not consider the growth of other organs, nor does it model facial growth or interactions among the skull base, calvarium, and face. The specific aim of the present study is to address these limitations by developing a more comprehensive and validated framework to predict the whole craniofacial growth (Figures 1 and 2). In this work, we have built upon our recent characterization of normal craniofacial growth based on *in vivo* clinical computed tomography (CT) images (*n* = 217, 0–48 months).^33^ In this previous study, we estimated the average skull morphology at specific ages to provide validation targets for the present physico-mechanical model. It is worth highlighting that by a physico-mechanical model here, we refer to a functional biomechanical model based on 3D geometries (reconstructed from CT images) that uses the FE method to take into account the interactions between developing constituents of the craniofacial system^13^^,^^28^ (Figures 2A–2D). This model provides a new framework to predict craniofacial growth from birth to 48 months of age (Figures 2E–2I and 3). Various sensitivity analyses regarding the choice of input parameters were carried out in the course of model development (see supplemental information). We validated its predictions by comparing them with our prior clinical *in vivo* measurements (Figure 4). We then used the proposed framework to comment on the changes in strain pattern across the skull and cranial sutures at specific ages during early ontogeny and biomechanical interactions between different elements of the craniofacial system (Figure 5). This biomechanical model coupled with a parameterized computational framework demonstrates great potential to investigate fundamental questions in developmental biology, i.e., the regulation of mechanical stimuli on growth processes in the development of cranial form, and to advance pre-surgical planning for craniofacial surgery e.g., on craniosynostotic patients.

## Results

### Sensitivity tests

We carried out 6 sensitivity tests including 16 independent analyses to optimize the key input parameters for the final model. These assessed the effects of varying material properties, boundary and interface conditions (Figure 2F), and growth simulation algorithms (Figures 2G and 2H; see full details of sensitivity tests in Figures S2–S9). These tests were conducted sequentially, and each test was designed to improve the prediction of the growth of one specific region through several case analyses. Validated configurations from initial sensitivity tests were used as the base case for subsequent sensitivity tests until all parameters were examined. The final results are presented below, and validated parameters are outlined in STAR★Methods.

### Predicted bone formation at the sutures

Our tissue differentiation algorithm (Figure 2H) predicted the pattern of bone formation in the craniofacial sutures. As shown in Figure 3, bone formed at the sutures and synchondroses at different pre-defined rates (Table 1), resulting in different closure timings (see STAR★Methods). By 12 months of age, all initial sutures/soft tissues components were differentiated into 9 newly formed layers of bone/hard tissues, with their elastic modulus ranging from 130 MPa to 930 MPa at 12 months of age. The elastic modulus of the bone at the initial age (i.e., 3 months of age) and 9 formed layers were updated by 125 MPa per month. This led to an elastic modulus of 5921 MPa for the bone and a range from 4630 MPa to 5430 MPa for the 9 differentiated tissue layers at 48 months of age. From available data in the literature, the elastic modulus of cranial bones gradually increases during postnatal development, to approximately 1265 MPa at 1–2 years of age^34^ and to around 6000–11000 MPa at 6 years^35^^,^^36^ over the calvarial region. The predicted elastic modulus of the cranial bones from the *in silico* model are in line with previously reported experimental data.

**Table 1:** Details of pre-defined specific closure rates of sutures, fontanelles and synchondroses

Component name	No. (Left, right)	Category	Dimensions of baseline model (mm)	Closure rate mm/month
Metopic suture	24	Early fused suture and fontanelles	6.1 (Mean width)	0.6^a^
Anterior fontanelle	40		33.7 ∗ 35.6 (Max. width∗length)	1.2^b^
Posterior fontanelle	41		17.6 ∗ 12.5 (Max. width∗length)	2^b^
Sagittal suture	27	Large calvarial sutures	7.6 (Mean width)	0.4^a^
Coronal suture	25,26		4.3 (Mean width)	0.2^a^
Lambdoid suture	28,29		3.5 (Mean width)	0.2^a^
Squamosal suture	30,31		2.8 (Mean width)	0.2^a^
Group of sutures, fontanelles and synchondroses in lower part of neurocranial region	32-39, 42-45	Small size or late fused components of lower part of neurocranial region	1.6–3.5 (Min. - Max. width; sutures) 5.1–6.9 (Min. - Max. width; fontanelles) 1.5–2.2 (Min. - Max. width; synchondroses)	0.2^a^
Group of sutures in midfacial region	46-54, 57-73	Small size or late fused sutures/fissures in midfacial region	1.1–2.3 (Min. - Max. width)	0.2^b^
Interior and superior orbital fissures	55,56		2.8–7.8 (Min. - Max. width)	0.2^b^

a Closure rates were sourced from Cross et al.^30^^,^^31^

b Closure rates were defined by authors.

### Measurements and comparison

Figure 4 presents the comparisons between the *in silico* predictions and *in vivo* data (*n* = 217). The expanded volumes differed less than 5% from the target volumes up to 48 months of age (Figures 4A–4D). The predicted cranial circumferences and cranial modules (CMs) based on the *in silico* models were in line with *in vivo* data (Figures 4E and 4F). Calvarial and midfacial shape changes during ontogeny followed the same trend as the *in vivo* data, as summarized by cephalic index (CI) and mid-facial index (MFI; Figures 4G and 4H). However, *in silico* models did not adequately simulate the growth of the zygomatic arches (Figure S10).

Figure 4I presents and compares the internal and external morphology of the *in silico* models and with the average skulls at 5 specific ages based on 2D cross-sections. From the mid-sagittal plane (P1), it is evident that the model predicted the growth of the entire cranium up to 48 months. The overall morphology of the predicted facial skeleton was consistent with the average models in the first 12 months of life. Nonetheless, there were obvious shape differences (>5 mm) in the orbital region and forehead (P2, Figure 4I) from 24 months onwards. The *in silico* models accurately predicted the lateral growth of the temporal-occipital region at skull base (P3, Figure 4I) and the antero-posterior displacement of the facial complex from 12 to 48 months (P4, Figure 4I).

A full comparison of the overall morphological differences between the external surfaces of *in silico* models and average skulls is shown in Figure 4J. We used histograms to quantify the degree of mis-match between the *in silico* and *in vivo* models. Results indicate that over approximately 80% of outer surface distance differences (mis-matches) between predicted and *in vivo* average models were within the range between −5 mm (under-prediction) and +5 mm (over-prediction). The greatest differences were in the posterior regions of the cranial vault (except the mid-sagittal region, fourth row in Figure 4J), with under-prediction in this region tending to increase with age (first row in Figure 4J). The areas of over-prediction varied with age. The model over-predicted the growth of the forehead (anterior part of cranial vault, first row in Figure 4J) and the posterior part of the skull base around 6 months of age, and the eminences became flatter up to 48 months of age (third row in Figure 4J). In some regions, the under/over-predictions improved over time, e.g., an excessive lateral expansion of the middle cranial fossa was observed up to 24 months of age, which then started to show a close match with the average skull beyond this point (third row in Figure 4J).

### Strain magnitudes and biomechanical interactions

Changes in overall mechanical strain (von Mises strain, Figure 5) and maximum tensile strain (first principal strain, Figure S12) across the craniofacial skeleton and suture components at the specific ages of 6, 12, 24, 36, and 48 months are reported. There are no obvious differences in the overall pattern of distribution and magnitude between these two strain regimes due to the size change (growth) of the internal organs and capsules dominating the postnatal ontogeny. These results highlight the dynamic shifts in the patterning of strains (both von Mises and first principal strains) across the calvaria, skull base, and face reflecting the differential growth rate of these regions.

During the first year of life, similar strain patterns were observed at 6 and 12 months, when calvarial growth was predominant driven by rapid brain expansion (sagittal view, Figure 5). This was reflected in the model by the greater magnitudes of von Mises strain in the calvarial sutures than those of skull base and midfacial regions. Calvarial sutures experienced highest von Mises strains up to 160000 με, almost eight times greater than the maximum von Mises strain over the adjacent calvarial bones at 6 months (superior view, Figure 5). From 12 to 24 months, the magnitudes of von Mises strain over the calvaria decreased while higher level of von Mises strain were observed over the skull base (especially the spheno-occipital synchondrosis) and the midfacial regions (anterior and inferior views, Figure 5). This occurred hand in hand with greater expansion of the midfacial complex, the sphenoid, and the occipital region relative to other regions. From 24 to 48 months, higher von Mises strain magnitudes were observed across the temporoparietal, orbital, and nasal regions (sagittal and anterior views, Figure 5). The temporoparietal region was under von Mises strain of around 30000 με at 24 months, and this level of von Mises strain gradually dissipated across the entire parietal bones and the lower occipital region with continued growth (sagittal view, Figure 5). Meanwhile, a high magnitude of von Mises strain (around 70000–80000 με) was observed within the nasal cavity, and adjacent maxilla had median levels of von Mises strains ranging from 10000 to 35000 με at 24 months (anterior view, Figure 5). The expansion of the midfacial complex peaked by 36 months, with von Mises strains over the nasal cavity being greater than 80000 με and the entire midface as well as palate experienced von Mises strains from 35000 to 65000 με (anterior and inferior views, Figure 5). Subsequently, elevated magnitudes of von Mises strain developed within the orbits and in the midfacial region at 48 months (i.e., ca. 32000 με) were observed.

## Discussion

We previously showed that calvarial growth is mainly driven by brain growth.^26^ Indeed, modeling isometric growth of the brain coupled with contact theory and a tissue differentiation algorithm at the calvarial sutures has previously provided excellent predictions of not only normal calvarial growth but also calvarial growth following its reconstruction e.g., in the case of sagittal craniosynostosis.^29^^,^^30^^,^^31^^,^^32^ In the present study we extend previous work^26^ by expanding key constituents/volumes of the facial region i.e., eyes, nasal cavity, and tongue (here modeled as upper intraoral volume, UIV) to assess the extent to which this enables us to predict whole craniofacial growth.

Our results indicate that the isometric expansion of these volumes is inadequate to predict cranial growth and highlight the crucial role of the skull base.^37^ When we incorporated the expansion of the skull base (Figures 2G and S7), we observed antero-posterior (sagittal) growth of the face but no dorsoventral (vertical) growth. However, when we also included expansion of maxilla, we then observed adequate dorsoventral growth of the face and its interplay with the orbits. These alterations of directional growth, together with the expansion of the skull base and maxilla, led to a surprisingly close match with our *in vivo* dataset (see Figure 4). These findings are consistent with current hypothesized mechanisms of craniofacial growth and highlight the importance of physical interactions.^2^^,^^6^^,^^13^^,^^18^

Our validated functional model of craniofacial growth predicted the magnitudes of mechanical strains that the craniofacial skeleton experiences during growth,^6^ and these predictions of dynamic increases and decreases in the levels of mechanical strains in different regions of the craniofacial system are consistent with known accelerations and decelerations of the growth in different regions.^3^^,^^33^ For example, the rapid calvarial growth in the first 12 months of life is mirrored in the high level of strains in the calvarial region (at both sutures and bones). These then gradually decrease from 12 months onwards. Subsequently, accelerations in the growth of the facial regions (NCV and UIV) lead to an increase in the magnitudes of mechanical strains that synchondroses and facial sutures experience. Nonetheless, full and detailed validation of the magnitudes of mechanical strain predicted here is challenging, not least because the strains arising in the model omit strains that result from the action of masticatory and neck muscles on the cranium.^15^^,^^16^ Further studies are required to improve this situation and achieve more accurate and comprehensive modeling of strains.

The computational framework described here enables us to predict craniofacial growth. This approach enables us to further investigate the mechanics of facial growth and how different surgical techniques might impact subsequent craniofacial growth. Thus, it opens up the possibility of exploring the consequences of different calvarial surgical techniques^31^ that may impact facial growth and so, of assessing the efficacy of different techniques in treating conditions that affect growth of the face. This is of particular importance, for example in treating syndromic forms of craniosynostosis where different surgical techniques have been developed to correct disturbed facial growth. Further work is required to test the application of this computational tool in these areas and to assess its limitations.

In summary, we have presented a physico-mechanical model of neonatal craniofacial system and have described a parameterized framework for modeling the growth of craniofacial regions up to 48 months of age. This framework permits testing current models of craniofacial growth and advances our fundamental understanding of the physics of these processes.

### Limitations of the study

The key limitations of the current computational framework are: (1) as a purely mechanical model with parameterized physical factors, we aimed to predict overall skull growth and to investigate the interplay among the bones and soft tissues at the macro-level while a combination of biological and chemical regulatory factors at the molecular and cellular levels are ignored. (2) The mandible with the masticatory muscles and the cervical spine with neck muscles were not considered in this framework. We believe they are key contributors to facial and skull base growth. In the present study their effects were modeled by the expansion of these regions (as outlined and tested through our sensitivity tests) but their omission might explain some of the observed mismatches e.g., at the zygomatic arch (Figure S10) and the posterior cranial vault (Figure 4J). (3) The eruption of teeth and their contribution to midfacial growth was not modeled, although the predicted models to some degree reflected the consequences of dental eruption on directional growth of the maxilla.

## STAR★Methods

### Key resources table

**Table:** 

REAGENT or RESOURCE	SOURCE	IDENTIFIER
**Deposited data**		

Craniometric data of normal individuals (*n* = 217, age: 0–48 months)	Liang et al.^33^	https://www.nature.com/articles/s41598-023-36646-8

**Software and algorithms**		

Avizo 3D Version 2022.1	Thermo Fisher Scientific Inc.	https://www.thermofisher.com/software-em-3d-vis/customerportal/download-center/amira-avizo-3d-installers/
HyperMesh Version 2022	Altair Engineering Inc.	https://altairone.com/Marketplace?tab=Info&app=HyperMesh
ANSYS Mechanical APDL Version 2022 R2	ANSYS Inc.	https://www.ansys.com/en-gb/products/structures/ansys-mechanical
Strain-based tissue differentiation algorithm	This paper; Marghoub et al.^28^	https://doi.org/10.1103/PhysRevLett.122.048103
Thermal analogy volume expansion algorithm	Libby et al.^26^	https://doi.org/10.1098/rsif.2017.0202

### Resource availability

#### Lead contact

Requests for further information and resources should be directed to and will be fulfilled by the lead contact, Prof. Mehran Moazen (M.Moazen@ucl.ac.uk).

#### Materials availability

This study did not generate new materials.

#### Data and code availability

• All data generated or analyzed during this study are included in the manuscript and supplementary tables and figures.
• The code (wrote using ANSYS Mechanical APDL commands) for the presented modeling approach can be accessed by reaching out to the lead contact. Besides, this approach can be replicated in ANSYS Workbench (graphic user interface) by following all the steps and parameters described in the STAR Methods section without using the APDL codes.
• Any additional information required to reanalyze the data reported in this paper is available from the lead contact upon request.

### Experimental model and study participant details

#### Ethic statement

Ethical approval was obtained for this study from Necker – Enfants Malades University Hospital under №2018RK18.

### Method details

#### *In vivo* characterization of the normal craniofacial growth

We obtained 217 anonymized head computed tomography (CT) stacks of normal children (94 females and 123 males) from 0 to 48 months of age from the Necker – Enfants Malades University Hospital, Paris (study No. 2018RK18). All these individuals were born between 2008 and 2018 and scanned for clinical purposes to investigate minor trauma, head and neck acute infections, or febrile seizures. Skull models were developed using Avizo image processing software (FEI V9.2., Thermo Fisher Scientific, Mass, USA) with mandibles being removed (Figure 2A). We previously created a normative *in vivo* dataset comprising a series of 2D and 3D measurements of all 217 individuals to characterize early craniofacial growth and development.^33^ In the present study, 12 linear dimensions and 4 cranial volumes (Figures 2B and 2C; Table S2) were included and used as input parameters for the physico-mechanical model of postnatal craniofacial growth or as control data for further comparison (see below). Mean values of four cranial volumes (i.e., Intracranial Volume (ICV), Orbital Volume (OV), Nasal Cavity Volume (NCV) and Upper Intraoral Volume (UIV)) at specific ages up to 48 months of age were estimated by regression analyses of the measured volumes of all individuals (Table S3A). Five skulls were selected from the *in vivo* dataset representing the ‘average’ model at each target age i.e., 6, 12, 24, 36 and 48 months (Figure 2D; Table S3B), according to their actual ages (±1 month of the target age), volumes of inner cranial cavities (<10% differences compared with mean values), and quality of reconstructed models (relatively complete skeleton structure and symmetric shape).

#### Baseline *in silico* model development

A female skull (individual F_111; Figure S1; Table S3B) that closely matched the average model at 3 months of age was used to develop the baseline FE model. The skull was initially reconstructed in Avizo, then manually segmented into 23 cranial bones, 40 sutures, 4 synchondroses, 6 fontanelles, 4 volumes (including paired orbital volumes) and 4 artificial covers (Figures 1A and S1), making several assumptions and simplifications (Table S1) to achieve a compromise between maintaining the details of anatomical structures during reconstruction^7^^,^^38^ and meeting the requirements of simulation.^13^^,^^39^^,^^40^ For example, all sinuses (i.e., frontal and maxillary sinuses) within the skeleton were filled and assigned the same material properties as bone. Also, the width of the sutures, fontanelle and synchondroses increased by 2–4 mm resulting in flat and smooth contact interfaces (i.e., sagittal suture in Figure S1), in preparation for the mesh generation and to avoid contact penetrations across the interfaces in the following simulations. The segmented geometric model was converted into a 3D solid mesh, consisting of approximately 5.3 million tetrahedral elements in total with varying mesh densities among different groups of components (∼1.3 million elements for cranial bones; ∼2.0 million elements for all sutures, fontanelles and synchondroses; ∼1.9 million elements for cranial volumes; ∼0.1 million elements for artificial covers; see Table S4 for details of mesh configurations and mesh size per group of components), modified in HyperMesh software (V2022, Altair Engineering Inc., Troy, Michigan, USA). Then, the meshed model was imported to a finite-element solver, ANSYS (V2022 R2, ANSYS Inc., Canonsburg, PA, USA).

#### Material properties

Isotropic (linear and elastic) material properties were assigned to all components. Bones (Figure 1A, No. 1–18), cartilages (Figure 1A, No. 19–23), and sutures (including synchondroses and fontanelles; Figure 1A, No. 24–73) were assumed to have a baseline elastic modulus (E) of 421 MPa, 100 MPa, and 30 MPa, and a corresponding Poisson’s ratio of 0.22, 0.22, and 0.3 at 3 months of age respectively.^26^^,^^30^^,^^31^^,^^40^ The E of ICV (Figure 1A, No. 74) was defined as 50 MPa,^41^ and the E of OV (Figure 1A, No.75-76) was lower, 10 MPa, because both eye sockets are occupied by soft tissues. The nasal cavity volume (NCV) comprises several cartilaginous and bony elements (i.e., vomer bone), various soft tissues and airway spaces. The upper intraoral volume (UIV) refers to the volume of the part of the oral cavity enclosed by the arch of the palate.^33^^,^^42^ Simulations of changes in these two volumes aimed to expand the relevant regions to the correct shape and size, and to predict the growth contributions of these soft tissues as well as brain (ICV) expansion (Figure S9). Hence, the baseline E of the NCV and UIV (Figure 1A, No.77-78) were set as 421 MPa. The foramen magnum cover (Figure 1A, No. 79) was allocated the same material properties as bony components while the other three covers (Figure 1A, No. 80–82) were allocated the same material properties as the sutures. The Poisson’s ratios of bone, cartilage, suture, volume, and cover components were assigned as 0.22, 0.22, 0.3, 0.1 and 0.1 respectively.^26^^,^^31^ Detailed descriptions of all components mentioned above can be found in Table S1.

#### Boundary and interface conditions

One-directional nodal displacement constraints were placed on facial component covers (Figure 1A, No. 80–82) as shown in Figure 2F. These were used to eliminate the expansion of the virtual covers due to the expansion of the volumes they overlaid i.e., eyes and upper palate (Figure S3). To minimize rigid displacement and avoid over constraining, nodal constraints with one degree of freedom (in the antero-posterior direction) were placed around the basilar portion of occipital bone, while one-directional nodal displacement constraints (in the dorsal-ventral direction) were placed around the foramen magnum (Figure 2F). Note that the values of displacement constraints on each component at each load-step were determined through sensitivity tests (Figures S4–S6). Penalty-based frictional contacts^31^ were established between four cranial volumes (ICV, OV, NCV and UIV) and inner-skeletal interfaces. A friction coefficient of 0.1 was applied to these contacts, where normal and tangential movements were granted and controlled during simulation. A normal penalty stiffness of 100 N/mm was attributed to the contact across ICV and OV and 600 N/mm was used for NCV and UIV, with the same penetration tolerance of 0.5 mm. These two values were defined to minimize interpenetrations among contacted surfaces. All of the above parameters were chosen based on previous studies.^29^^,^^30^^,^^31^^,^^32^ The bone-suture interfaces were perfectly bonded i.e., no relative normal and tangential movement was allowed.^26^^,^^28^

#### Modeling craniofacial growth

Brain growth and the expansion of midfacial cavities^33^ were modeled using a thermal analogy algorithm.^27^^,^^29^ In this study, ICV, OV, NCV and UIV components were expanded to the target values (i.e., mean values) from 3 months to 48 months of age in 15 load-steps (Table S3) by defining radial (α_xyz_) or directional (α_x_, α_y_, α_z_) expansion coefficients (Figure 2G). Input expansion coefficients that led to less than a 5% difference between the predicted volume and the target value at each load-step were considered acceptable. Additionally, selected components of the skull base (sphenoid, temporal bone, and basilar and lateral portions of occipital bone) and midfacial region (upper portion of ethmoid bone, lateral wall of nasal cavity, zygoma, and maxilla) were expanded radially and directionally (Figure 2G) to simulate, in a simple way, the deposition and resorption of bones during early growth.^2^ Several sensitivity tests were carried out on the choice of various input parameters used in modeling the expansion of cranial volumes, skull base and midfacial components, and these are summarized in the Figures S7–S9.

#### Modeling bone formation at the sutures

A strain-based tissue differentiation algorithm^28^ was implemented at each load-step to model bone formation at the sutures (including fontanelles) and synchondroses. Considering the various rates and timings of suture closure in the first years of life, we defined different closure rates for sutures based on the literature,^7^^,^^8^^,^^9^ our previous studies^28^^,^^31^ and the size of segmented sutures in the baseline model (Table 1). The metopic suture, anterior, and posterior fontanelles were assumed to have a bone formation rate of 0.6, 1.2 and 2 mm/month respectively, considering their early fusion. The sagittal suture was assumed to have a bone formation rate of 0.4 mm/month. The other calvarial sutures (including coronal, lambdoid, and squamosal sutures), all facial sutures, and synchondroses were assumed to have a bone formation rate of 0.2 mm/month.

The tissue differentiation algorithm selected the tissue to be differentiated in two steps (Figure 2H): first, sutural elements with hydrostatic strain in the range of 0–5% were selected according to previous studies^28^^,^^43^ and sensitivity tests (Figure S11); second, the elements with a predefined radius from the edge of the previously formed tissue (equal to bone formation rate multiplied by age intervals of one load-step) were re-selected from the initial selection. The final selected elements/tissue were attributed to a new component representing newly formed tissue. Then, to replicate the differentiation of bone stiffness^28^^,^^31^ and to prepare for the next load-step, the skull geometry was updated to the new deformed shape and the elastic moduli of pre-existing bone, newly formed tissue, cartilage, and cover components were increased by 125 MPa/month, 100 MPa/month, 100 MPa/month, and 100 MPa/month, respectively (Figure 2I). The choice of 125 MPa/month and 100 MPa/month for pre-existing bone and newly formed tissue was based on a prior study replicating tissue differentiation in normal and syndromic human models,^16^^,^^32^ while the increase in the moduli of elasticity of cartilage was assumed to be a lower rate (100 MPa/month) than the bony tissues. The stiffening across the cover components was same as that of connective tissues to accommodate to the expansion of enclosed volume/cavity.

#### Morphological analysis

We compared the predicted craniofacial morphology resulting from these simulations with *in vivo* data. Here, size and shape measurements, cranial circumference and three cranial indices (cranial module - CM, cephalic index - CI, and mid-facial index - UFI) derived from several linear dimensions (Figure 2B) based on the *in vivo* data^33^ were used for the comparison. The major shape differences were highlighted by aligning the 2D cross-sections of the *in silico* models and *in vivo* average skulls at 6, 12, 24, 36 and 48 months in four planes (one mid-sagittal, two coronal, and one transverse planes; Figure 1B). The overall morphological differences (in mm) between the external surface of the *in silico* models and *in vivo* skulls at these ages were visualized and quantified using 3D distance plots.^26^ The 2D cross-sections and 3D distance plots were created in Avizo and based on the alignments shown in Figure 1B.

## Supplemental information

Document S1. Figures S1–S12 and Tables S1–S4

Figure360. An author presentation of Figure 1
