# Supplementary material for: A physico-mechanical model of postnatal craniofacial growth in human
Source: iScience. 2024 Jul 29;27(9):110617. doi: 10.1016/j.isci.2024.110617 (PMC11365398; doi:10.1016/j.isci.2024.110617)
Supplement: Document S1. Figures S1–S12 and Tables S1–S4 [file mmc1.pdf]

iScience, Volume *27*

## Supplemental information

# A physico-mechanical model

# of postnatal craniofacial growth in human

**Ce Liang, Arsalan Marghoub, Antonio Profico, Costantino Buzi, Marius Didziokas, Lara van de Lande, Roman Hossein Khonsari, David Johnson, Paul O'Higgins, and Mehran Moazen**

**This PDF file includes:**

Figures S1 to S12

Tables S1 to S4

SI references [S1-S7] (same with refs [7-9, 26, 27, 31, 33] in the Main Text)

## Supplemental Figures

**Figure S1**. Visualization of the final *in silico* baseline model and initial reconstruction of *in vivo* skull from a female individual at around 3 months of age: **(a)** 3D views. **(b)** 2D cross-sections in four planes. Note that details of the separated components of *in silico* model and the planes used for cross-sections are described in Figure 1 in the main text. This figure is related to Figure 1.

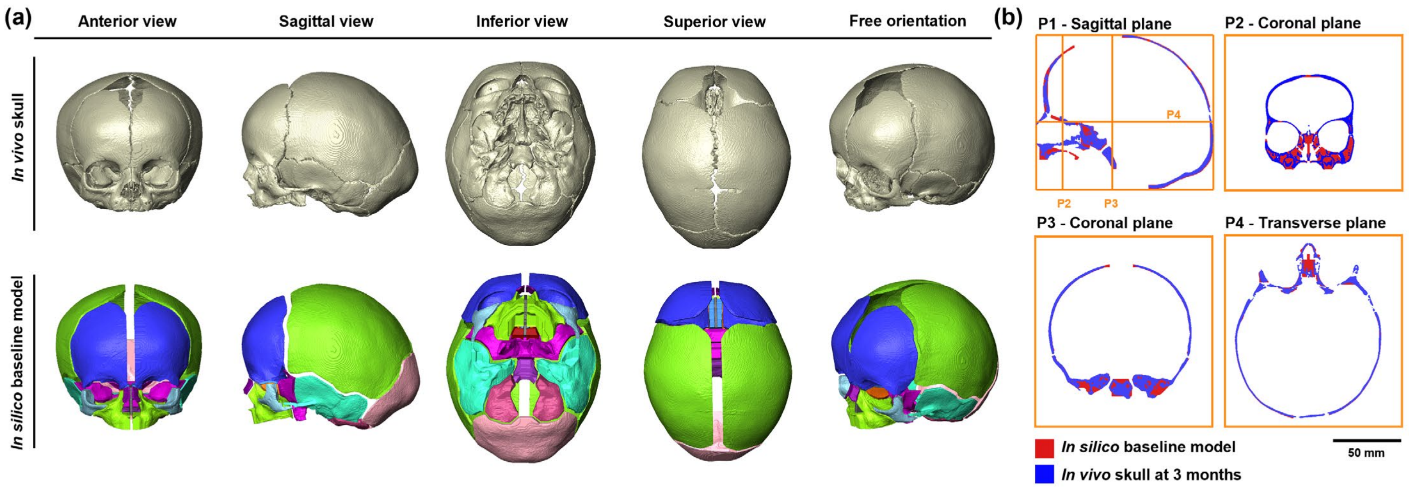

**Figure S2**. Flowchart of six sensitivity tests including 16 independent analyses carried out in this study, where the main method (left side) and major improvement from each test (right side) are presented. Each sensitivity test was carried out to improve the predictions at one specific region through several case tests (see Figure S3-S9). The validated configurations or parameters from the former test (in yellow) were used as the base case for the next test. The optimized configurations from the sensitivity test 6 – Case 2 were used for final simulations. This figure is related to STAR Methods.

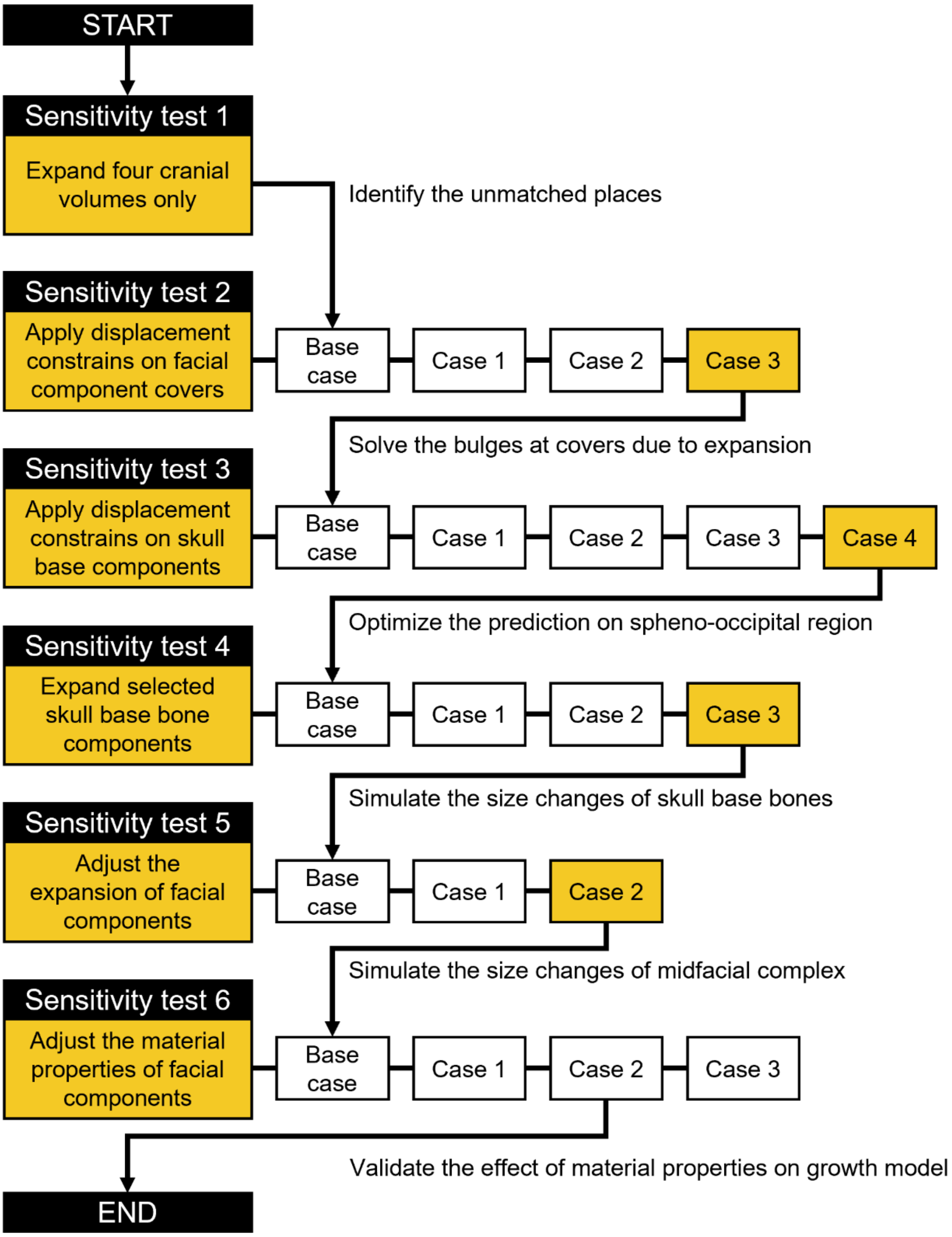

**Figure S3**. Overview of sensitivity test 1. **(a)** This test was to investigate the contribution of pure radial expansion ($\alpha_{xyz}$) [S1] of four cranial volumes (intracranial volume-ICV, orbital volume-OV, nasal cavity volume-NCV and upper intraoral volume-UIV) in modeling the postnatal craniofacial growth up to 48 months of age. Nodal constraints in all degrees of freedom were placed around the foramen magnum, as in prior studies [S1,S2], and bone formation at the sutures was modelled using the tissue differentiation algorithm described in STAR Methods. **(b)** 2D morphological comparison of inner skeleton between pre-aligned *in silico* models and average skulls at 6, 12, 24, 36 and 48 months of age using cross-sections from three planes. **(c)-(f)** Absolute differences between the predicted four cranial volumes and target values at each age. The results show the well-predicted calvarial growth (see sagittal plane in (b)) and volumetric changes (c), but less satisfactory predictions across the face and skull base, these include: (1) unexpected volume expansions displayed as bulges at facial component covers (OVC, NCVC and UIVC); (2) the differences in size and position of the spheno-occipital region between *in silico* models and the average *in vivo* individual became significant after 12 months (see sagittal plane in (b)); (3) a lack of prediction of skull base growth (i.e. temporal-occipital region; see coronal plane in (b)) after 12 months of age; (4) obvious mismatch of the entire midfacial complex after 6 months of age, including less growth of the nasal cavity and upper palatal region in the supero-inferior direction, and insufficient antero-posterior growth of the upper facial region (see sagittal and transverse planes in (b)). All insufficient predictions identified in this test were improved by the following tests, where sensitivity test 2 addresses problem (1), sensitivity test 3 addresses problem (2), sensitivity test 4 addresses problem (3), and sensitivity test 5&6 address problem (4). This figure is related to Figure S2 (sensitivity test 1) and STAR Methods.

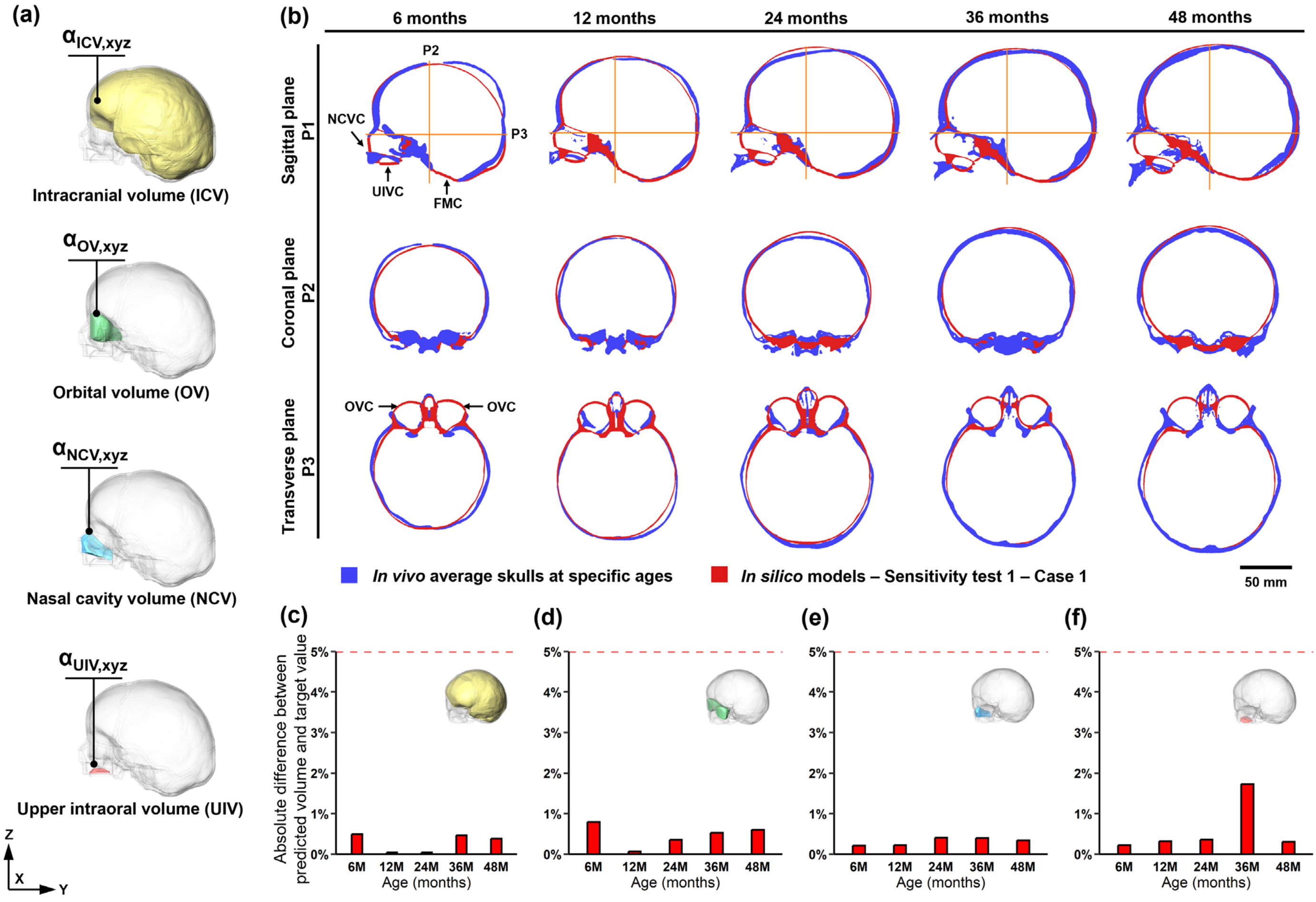

**Figure S4**. Measurements of eight linear dimensions used as references for the values of nodal displacement constraints applied in sensitivity tests 2 and 3: **(a)** six average skulls with three planes defined for measurements, and two methods of calculating the values of displacement (D) at each load-step (age) derived from linear measurements. D1-D2 measured in the mid-sagittal plane between two aligned skulls (different ages) indicate relative anterior movement of the nasal complex. Similarly, D3-D4 and D5-D6 measured in the sagittal and transverse planes indicate relative anterior growth of the orbital rim. D7 was measured to quantify palatal movement. **(b)** D1-D8 measured based on Method 1: the maximum values of D1-D8 between the 3 and 48 months of age skull models were measured from the grid plot, and the average displacement for each month were calculated through linear interpolation of the total 45 months of intervals. **(c)-(g)** D1-D8 measured for method 2: the maximum values of D1-D8 between each of the two skull models at target ages (i.e. between 3 and 6 months) were measured from the corresponding grid plot, then the average value was calculated through the linear interpolation of the corresponding age interval (i.e. 3 months). This figure is related to Figure S5 and STAR Methods.

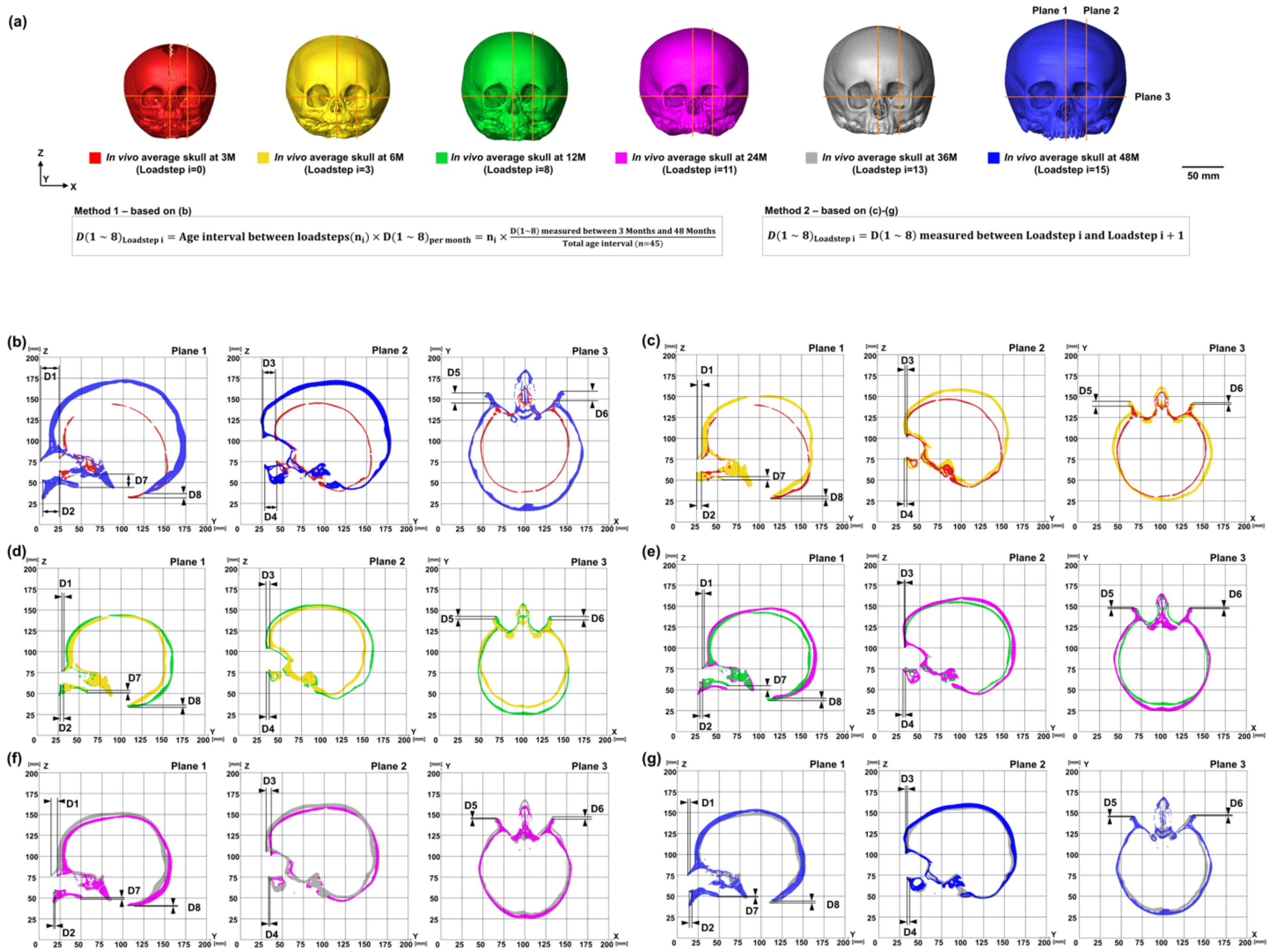

**Figure S5**. Overview of sensitivity test 2. **(a)** This test further expanded on sensitivity test 1 (Base case) by applying additional one-directional nodal displacement constraints to facial component covers (Case 1-3) to limit the undesirable volume expansion at covers (see 2D diagrams). Four local coordinate systems (in parallel with the global coordinate system) were created at the center of the external surface of each cover. Nodes on the external surface of each cover were selected and their coordinates were transformed to corresponding local coordinate systems for initialization (see 3D view). Displacement constraints in one specific direction were placed on selected nodes at the beginning of each load-step. The values of maximum nodal displacement applied to different covers in each load-step were characterized based on Method 1 (Figure S4) for Case 1, and Method 2 (Figure S4) for Case 2. In Case 3, the values for one-directional displacement constraints were optimized and defined through iterative tests at each load-step until no additional deformation of covers was observed (less than 10% changes in cover thickness). **(b)** 2D morphological comparison of inner and outer craniofacial skeleton between pre-aligned *in silico* models from all cases and average skulls at 6, 12, 24, 36 and 48 months of age shown as plots of cross-sections from three planes. **(c)-(f)** Absolute differences between predicted four cranial volumes from all cases and target values at each age. The results show that, by applying the nodal displacement constraints to facial component covers (OVC, NCVC and UIVC), there are no bulges observed at the covers due to the radial volume expansion (see (b)). Under the same expansion coefficients of four cranial volumes at each load-step from sensitivity test 1, the estimated values of displacement constraints applied in Case 1 and 2 led to significant changes in the predicted ICV, OV and UIV (see (c)-(e)), while Case 3 limited expansion of upper palate (see (f)). This figure is related to Figure S2 and STAR Methods.

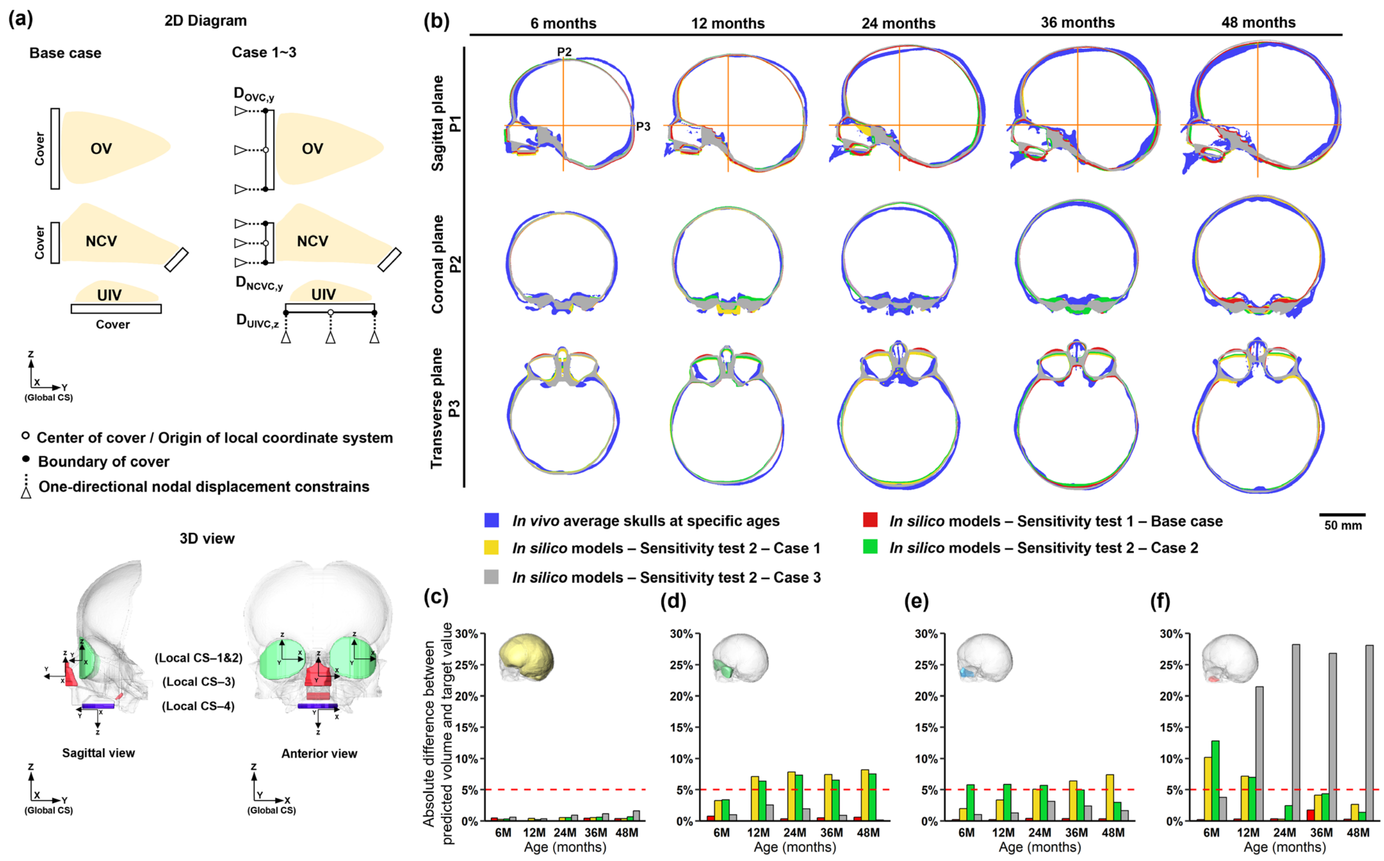

**Figure S6**. Overview of sensitivity test 3. **(a)** This test further expanded on sensitivity test 2 – Case 3 by altering nodal constraints on the skull base components (including sphenoid-S, basilar and later parts of occipital bone-O(b) and O(l)) to optimize prediction in the spheno-occipital region. This was carried out through four case tests: (1) Case 1 – Nodal constraints in all degrees of freedom (DOF) were placed around the basilar portion of the occipital bone; (2) Case 2 – Nodal constraints in all DOF were placed around the presphenoid region, following a prior study [S3]; (3) Case 3 – Nodal constraints in one DOF were placed around the presphenoid region, while the one-directional displacement constraints (which allow a certain degree of displacement of constrained nodes in one direction) were placed around the foramen magnum. The foramen magnum displacement values ($D_{O(l)}$) at each load-step were determined through iterative tests based on the measurement of D8 (see Figure S4), following the same method in sensitivity test 2 – Case 3; Case 4 – Nodal constraints in one DOF were placed around the basilar portion of the occipital bone with the same one-directional nodal displacement constraints placed around the foramen magnum. **(b)** 2D morphological comparison of inner skeleton between pre-aligned *in silico* models from all cases and average skulls at 6, 12, 24, 36 and 48 months of age using cross-sections from three planes. **(c)-(f)** Absolute differences between predicted four cranial volumes from all cases and target values at each age. The results show that, nodal constraints in all DOF placed on the basilar portion of occipital bone (Case 1) and presphenoid region (Case 2) led to excessive local deformation of the relevant bones, while rotation of the spheno-occipital region was observed in both case tests compared with the Base Case (see (b)). By combining the one-directional nodal displacement constraints on the foramen magnum with either nodal constraints in one direction on the presphenoid region (Case 3) or on the basilar portion of the occipital bone (Case 4), the excessive local deformation due to over constraining was eliminated (see (b)). The predicted cranial volumes in Case 4 are closer to the target volume than the others (see (c)-(f)). This figure is related to Figure S2 and STAR Methods.

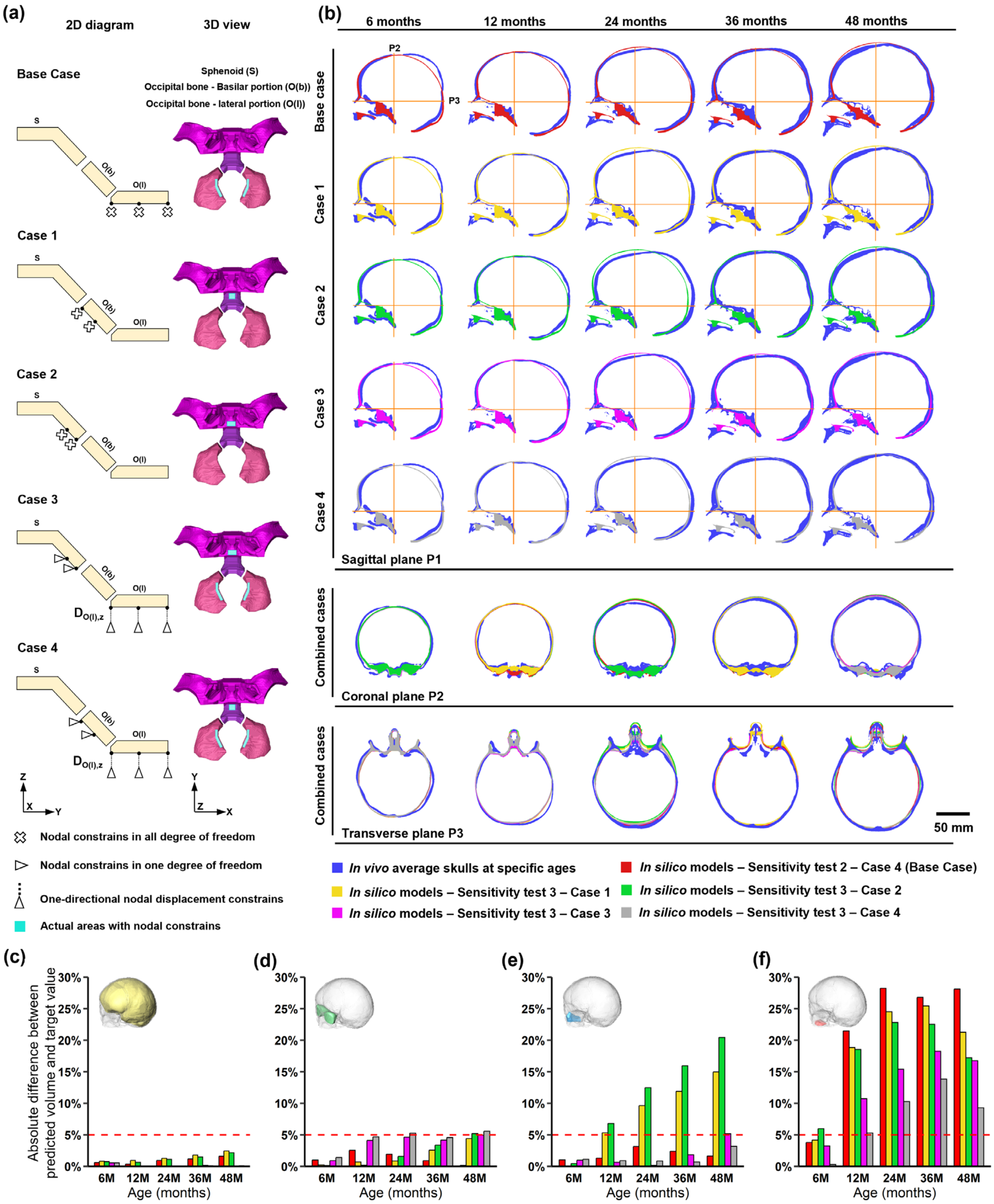

**Figure S7**. Overview of sensitivity test 4. **(a)** This test further expanded on sensitivity test 3 – Case 4 by applying additional ($\alpha_{xyz}$) or directional ($\alpha_x$, $\alpha_y$, $\alpha_z$) expansion to skull base components, including the sphenoid-S, temporal bone-T, basilar and lateral parts of occipital bone-O(b) and O(l), to optimize the prediction of the entire skull base region. This was carried out through three case tests: (1) Case 1 – S, T, O(b) and O(l)) were assumed to expand radially, and the expansion coefficients for each bony component ($\alpha_{S,xyz}$ ,$\alpha_{T,xyz}$ ,$\alpha_{O(b),xyz}$ and $\alpha_{O(l),xyz}$) were determined through iterative tests at each load-step, where the radial expansion coefficient was set to 0.1% at the beginning and then increased by 0.05% at each iteration, until the overall predicted shape matched the reference models at the skull base; (2) Case 2 – The sphenoid was assumed to expand directionally, more in the Y direction (antero-posterior direction) than in the X and Z directions (medio-lateral direction and superior-inferior direction). $\alpha_{S,y}$ was set at 0.5% while $\alpha_{S,x}$ and $\alpha_{S,z}$ were both set at 0.1% at the beginning with the same increment rate (0.05%) at each iteration (other configurations were the same as Case 1); (3) Case 3 – The sphenoid was assumed to expand in Y and Z directions (other configurations were the same as Case 1). **(b)** 2D morphological comparison of inner skeleton between pre-aligned *in silico* models from all cases and average skulls at 6, 12, 24, 36 and 48 months of age using cross-sections from three planes. **(c)-(f)** Absolute differences between predicted four cranial volumes from all cases and target values at each age. **(g)-(j)** Volumes of bone components of all cases measured at each age. The results show that, by applying additional radial expansion to the skull base bones, Case 1 over predicts growth of the sphenoid and temporal bones in the medio-lateral direction, while Cases 2 and 3 show good predictions of the overall size of the sphenoid (see sagittal plane in (b)). When no medio-lateral expansion is applied to sphenoid, Case 3 shows better prediction of spheno-temporal regions (see coronal plane in (b)). This figure is related to Figure S2 and STAR Methods.

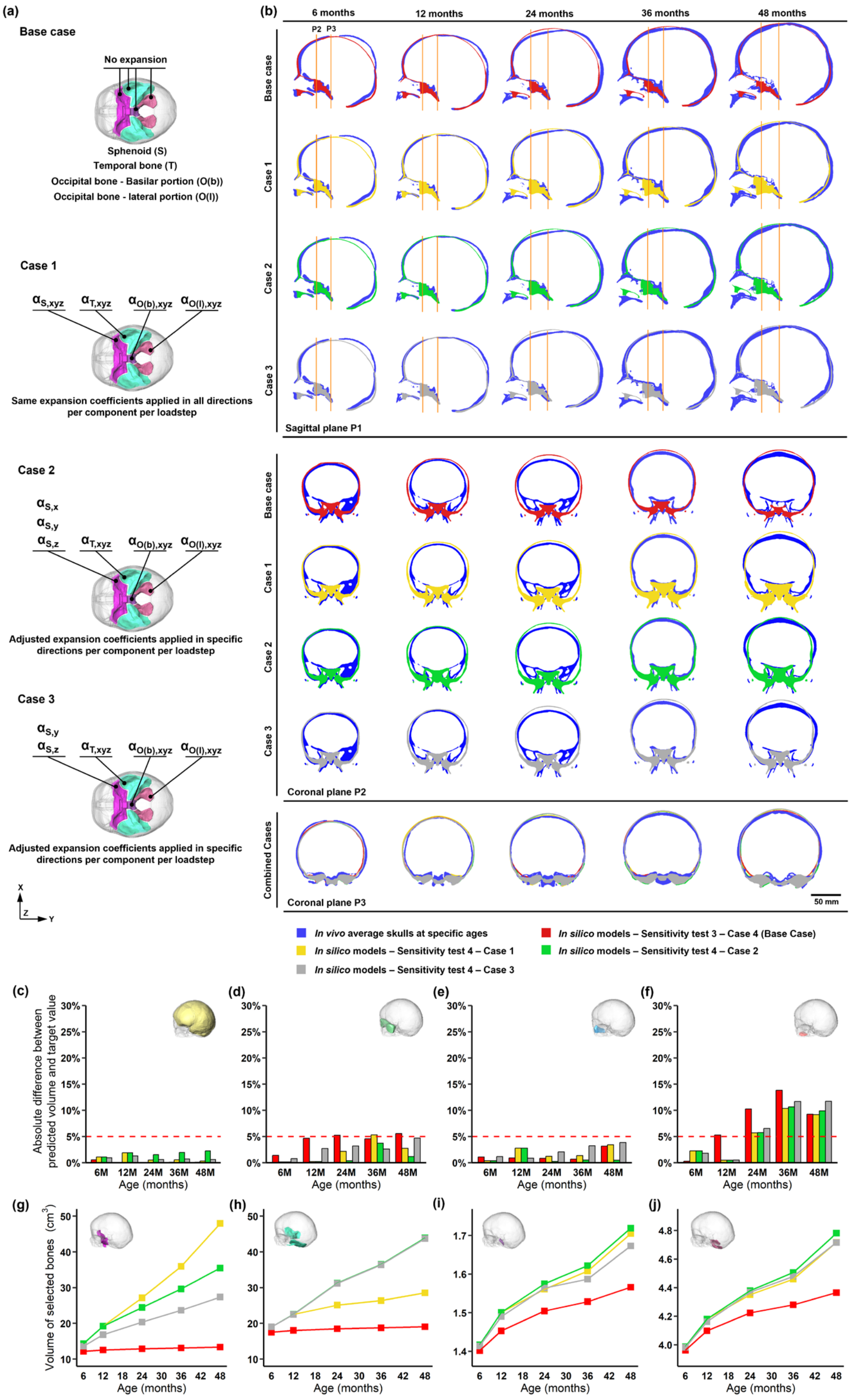

**Figure S8**. Overview of sensitivity test 5. **(a)** This test further expanded on sensitivity test 4 – Case 3 by applying additional ($\alpha_{xyz}$) or directional ($\alpha_x$, $\alpha_y$, $\alpha_z$) expansion to facial components, to optimize the prediction of the midfacial region. This was carried out through two case tests: (1) Case 1 – Directional expansion applied to NCV and UIV; (2) Case 1 – Along with the directional facial volume expansion in Case 1, four facial bony components (upper portion of ethmoid bone–E(u), lateral wall of nasal cavity-LN, zygoma-Z and maxilla-M) were further expanded radially or directionally. Similar to sensitivity test 4 (Figure S7), the expansion coefficient of selected facial components was determined through iterative tests at each age (load-step), where radial or directional expansion coefficients were set at 0.1% and increased by 0.05% at each iteration until the predicted OV, NCV and UIV reached target values, and differences were less than 5% between predicted facial dimensions (upper facial height and biorbital breadth) and mean values from the normative dataset (n=217). **(b)** 2D morphological comparison of inner skeleton between pre-aligned *in silico* models from all cases and average skulls at 6, 12, 24, 36 and 48 months of age using cross-sections from three planes. **(c)-(f)** Absolute differences between four predicted cranial volumes from all cases and target values at each age. **(g)-(j)** Volume of bone components of all cases measured at each age. The results show that, the directional expansion of NCV and UIV in Case 1 led to better predictions of the nasal cavity region (see sagittal plane in (b)), but the size difference in the midfacial complex between predictions and the average models becomes prominent after 12 months of age (see coronal plane in (b)). In Case 2, radial and directional expansion of key facial components optimized the prediction of midfacial growth and the resulting facial shapes presented a better match to the average models up to 48 months of age (see sagittal and coronal planes in (b)). This figure is related to Figure S2 and STAR Methods.

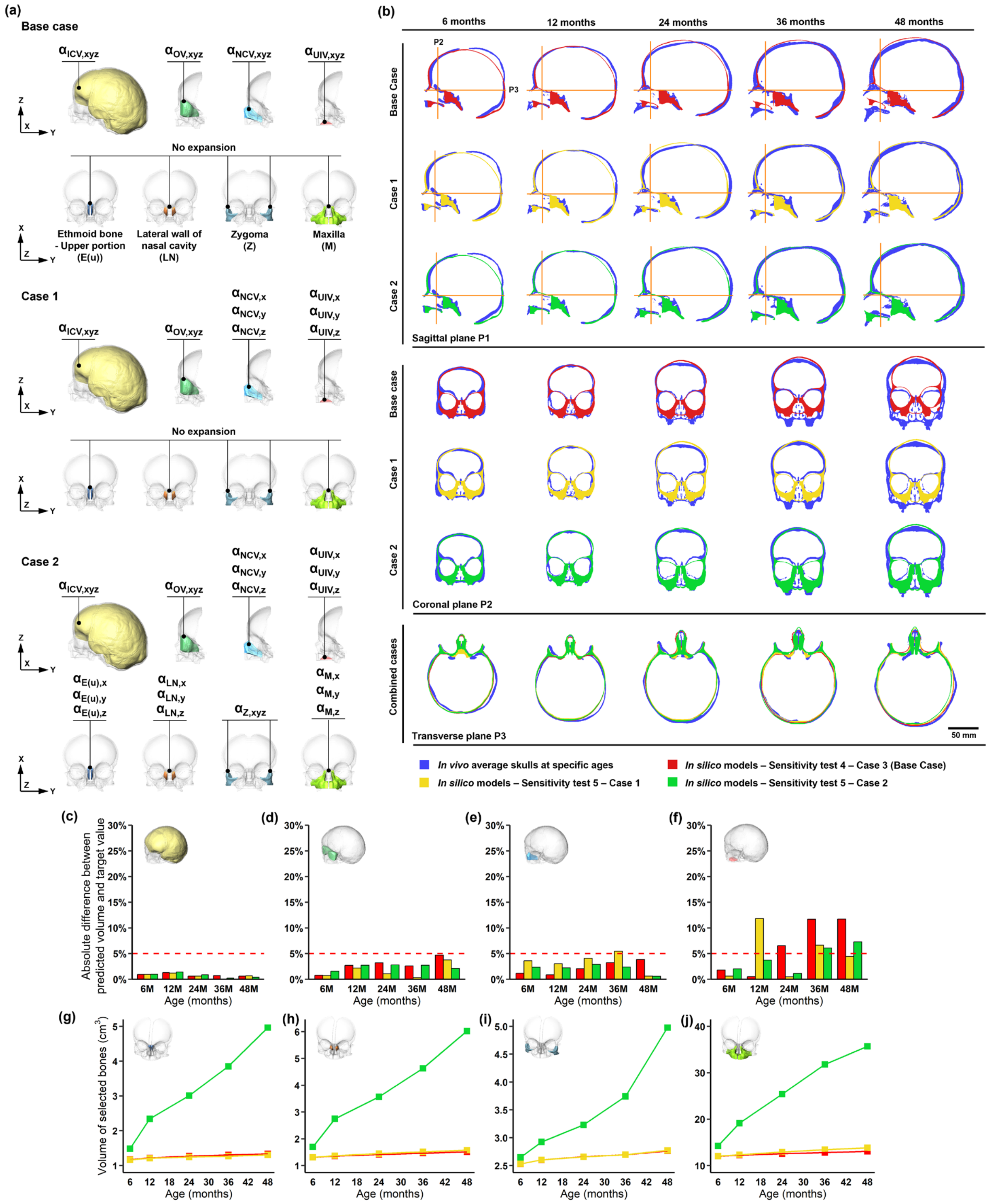

**Figure S9**. Overview of sensitivity test 6. **(a)** This test further expanded on sensitivity test 5 – Case 2 by altering the elastic modulus of NCV and UIV (referring to the volume of each defined space or cavity) to test the effect of material properties on predicted shapes. This was carried out through three case tests: (1) Case 1 – The elastic modulus (E) of NCV and UIV were set as 30MPa at 3 months of age (load-step 0) with an increment rate of 100 MPa/month (same as the material properties of suture components); (2) Case 2 – The E of NCV and UIV were set as 421MPa at 3 months of age (load-step 0) with E increased by 125 MPa/month (same as the material properties of bone components); (3) Case 3 – The E of NCV and UIV to 3000MPa at 3 months of age (load-step 0) with E increased by 125 MPa/month (an arbitrary extreme-large value of elastic modulus). Note that the expansion coefficients were adjusted to accommodate changes in material properties. **(b)** 2D morphological comparison of inner skeleton between pre-aligned *in silico* models from all cases and average skulls at 6, 12, 24, 36 and 48 months of age using cross-sections from four planes. **(c)-(f)** Absolute differences between four predicted cranial volumes from all cases and target values at each age. The results show that the difference in elastic modulus of NCV and UIV leads to non-significant effects on predicted facial shapes at specific ages. As expansion of these two volumes aims to expand the relevant regions to the correct shape and size rather than predicting the growth contributions of soft tissues such as the brain (intracranial volume), the configurations applied in Case 2 were used in final simulations. This figure is related to Figure S2 and STAR Methods.

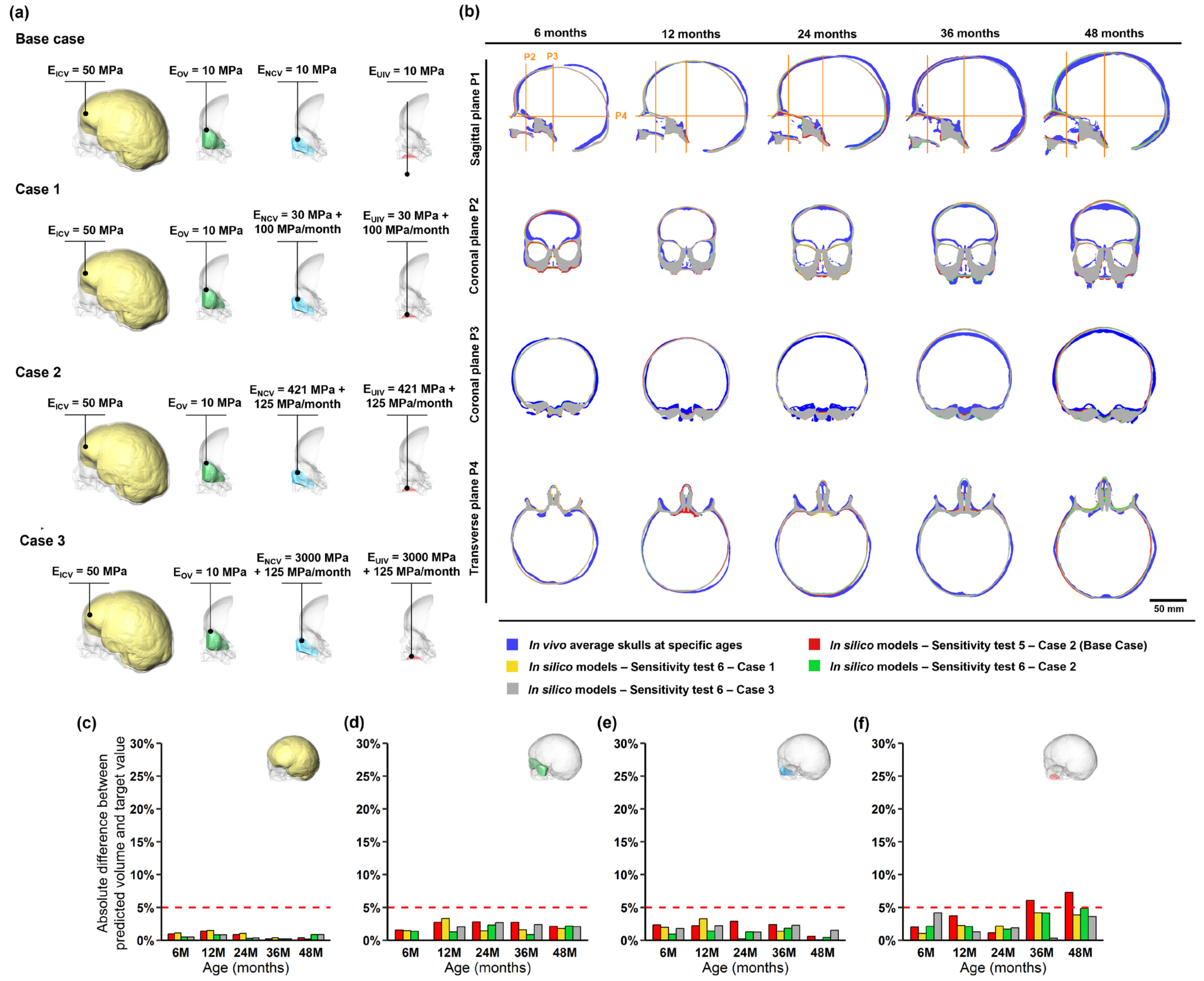

**Figure S10**. Scatter plots of the comparisons of the measurement of upper facial index (Upper facial height / Bizygomatic breadth x 100) between *in silico* models (in red) and *in vivo* average skulls (in blue) at 6, 12, 24, 36, and 48 months of age. Note that the corresponding data from the control group of 217 normal individuals (in grey) are also plotted in this scatter plot, indicating the overall changes and distributions in the normative dataset. This figure is related to Figure 4.

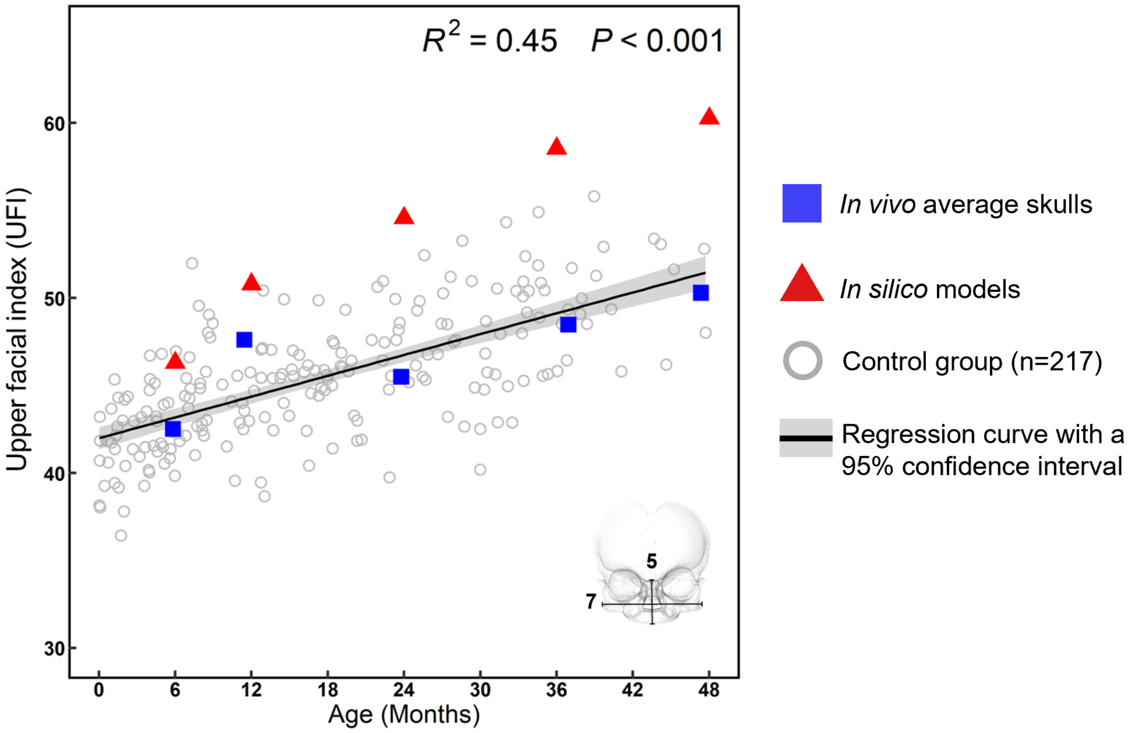

**Figure S11**. Sensitivity tests of hydrostatic strain range. 3D model highlights the overall hydrostatic strain across the *in silico* model after a single growth load-step from 3 to 4 months of age. The dashed box (in blue) shows the most applicable threshold of hydrostatic strain for this study. Note that regions shown in grey represent regions below or above the specified thresholds. This figure is related to STAR Methods.

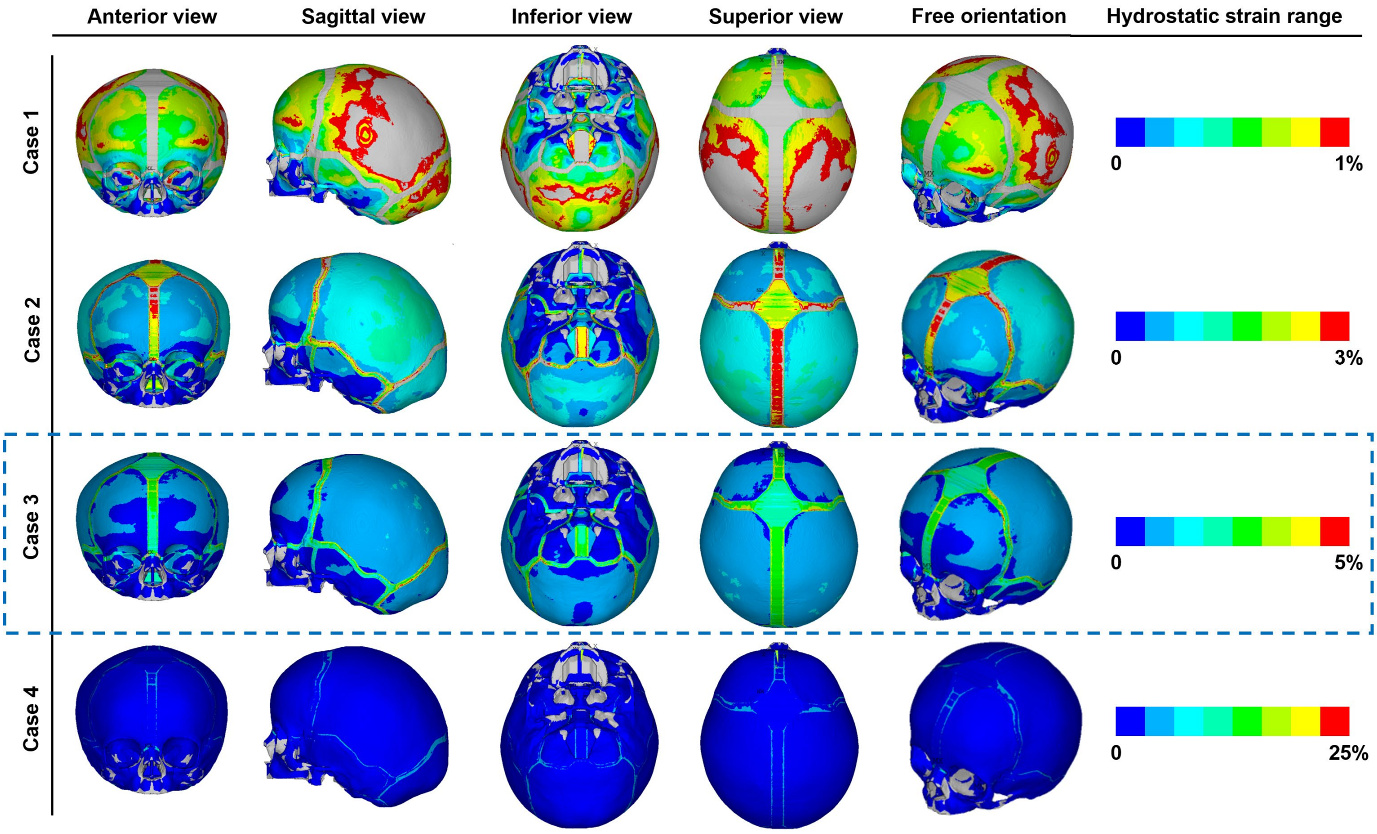

**Figure S12.** Changes in strain pattern across the skeleton and craniofacial sutures at 6, 12, 24, 36 and 48 months of age during growth. The first principal strains (1st με) were derived from one load-step between the former age to the target age and mapped onto the *in silico* model at the target age. Skeleton and suture components were scaled individually with different ranges of strain before 12 months representing both bone formation at sutures and tissue differentiation, then the same strain range was shared by skeleton and suture components up to 48 months. This figure is related to Figure 5.

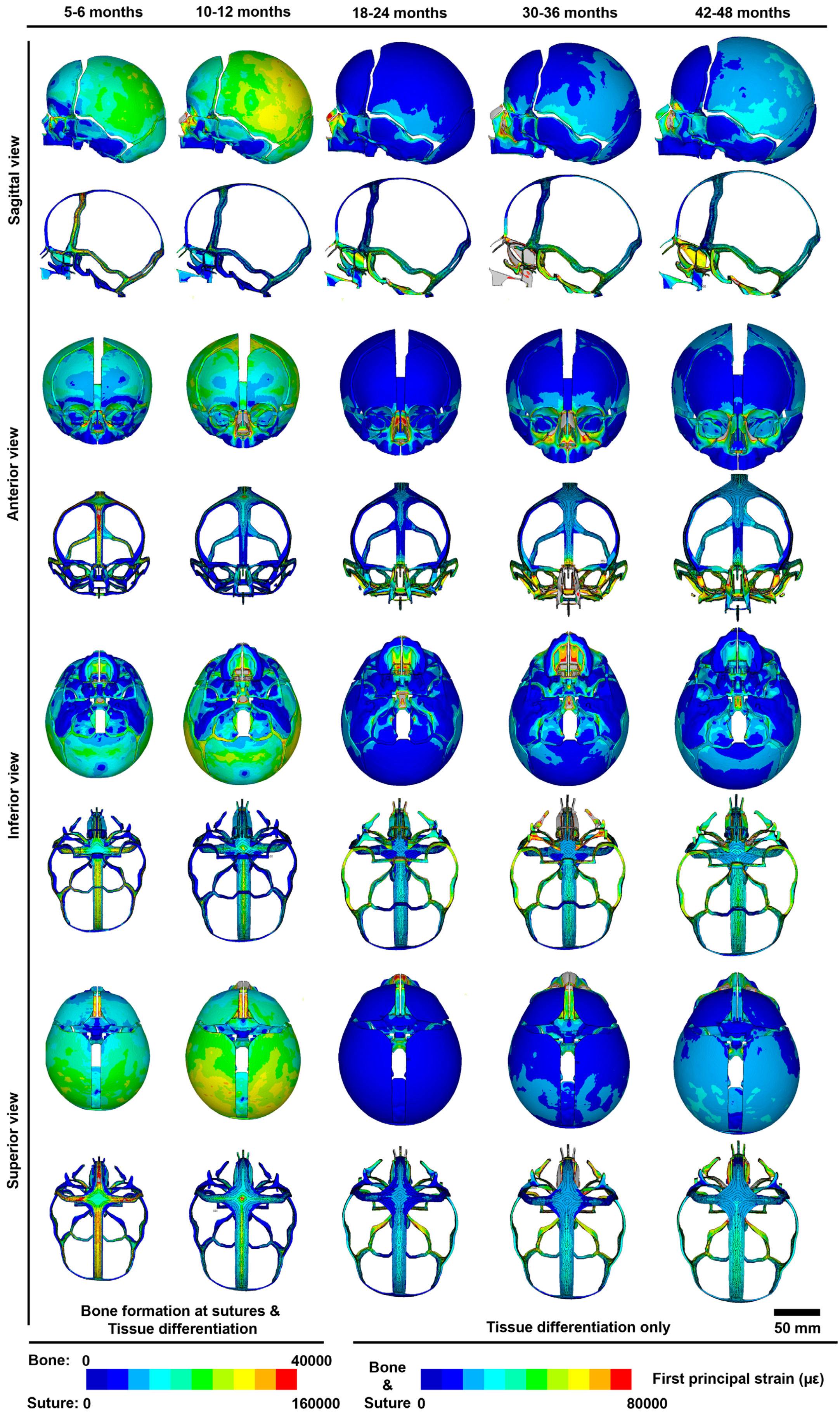

## Supplemental Tables

**Table S1.** Details of *in silico* baseline model including definitions of each component, abbreviations, and the assumptions or simplifications made during model development. This table is related to Figure 1.

| Component | No. (left, right) | Abbreviation | Category | Region | Assumptions or simplifications made during baseline model development |
|---|---|---|---|---|---|
| Frontal bone | 1,2 | F | Bone and Cartilage | Neurocranium | No frontal sinus found at the age of this baseline model (3 months), and frontal bones were filled and assigned the same material properties as bone. |
| Sphenoid | 3 | S | | | No sphenoid sinus found at the age of baseline model (3 months), and all sphenoid was filled and assigned the same material properties as bone. |
| Parietal bone | 4,5 | P | | | / |
| Temporal bone | 6,7 | T | | | All holes or sinuses were filled and assigned the same material properties as bone. |
| Occipital bone - Squamous portion | 8 | O(sq)[a] | | | / |
| Occipital bone - lateral portion | 9 | O(l)[a] | | | / |
| Occipital bone - Basilar portion | 10 | O(b)[a] | | | / |
| Zygomatic bone | 11,12 | Z | | Viscerocranium | / |
| Maxilla[b] | 13,14 | M | | | The anatomical structure of the lacrimal bone was simplified and combined in the current component. Infraorbital foramen, maxillary sinuses, and alveolar bones were filled and assigned the same material properties as bone. No teeth segmented or created in this model. |
| Horizontal portion of palate bone | 15,16 | HP[a] | | | / |
| Nasal bone | 17,18 | N | | | / |
| Ethmoid bone - Upper portion[b] | 19,20 | E(u)[a] | | | The anatomical structure of cribriform plate, crista galli, ethmoidal labyrinths and superior concha are simplified. Ethmoidal sinuses (air cells) were filled and assigned the same material properties as bone. |
| Lateral wall of nasal cavity[b] | 21,22 | LN[a] | | | The anatomical structures of orbital plate and middle nasal concha were simplified and combined in current component. |
| Nasal septum[b] | 23 | NS | | | The anatomical structures of the perpendicular plate of ethmoid bone, vomer bone and septal cartilage were simplified and combined in current component. |
| Metopic suture | 24 | ms | Suture, Fontanelle and Synchondrosis | Neurocranium - Upper part | Segmented at the same sutural width. |
| Coronal suture | 25,26 | cs | | | The overlapped parts between frontal and parietal bones were simplified. |
| Sagittal suture | 27 | ss | | | The overlapped parts between parietal bones were simplified. |
| Lambdoid suture | 28,29 | ls | | | The overlapped parts between parietal and occipital bones were simplified. |
| Squamosal suture | 30,31 | sqs | | | / |
| Sphenosquamosal suture | 32,33 | sps | | Neurocranium - Lower part | / |
| Spheno-occipital synchondrosis | 34 | sosc[a] | | | / |
| Occipitomsatoid suture | 35,36 | oms | | | / |
| Anterior intraoccipital synchondrosis | 37,38 | aisc[a] | | | / |
| Posterior intraoccipital synchondrosis | 39 | pisc[a] | | | / |
| Anterior fontanelle | 40 | af | | Neurocranium - Upper part | Overall shape has been simplified to be symmetric in mid sagittal plane. |
| Posterior fontanelle | 41 | pf | | | Overall shape has been simplified to be symmetric in mid sagittal plane. |
| Sphenoidal fontanelle | 42,43 | sf | | | / |
| Mastoid fontanelle | 44,45 | mf | | Neurocranium - Lower part | / |

| | | | | | |
|---|---|---|---|---|---|
| Spheno-ethmodial-maxillary suture[b] | 46 | sems[a] | | Viscerocranium | Segmented as a plate between sphenoid bone and midfacial complex. |
| Sphenofrontal suture | 47,48 | sfs[a] | | | / |
| Frontozygomatic suture | 49,50 | fzs | | | / |
| Sphenozygomatic suture | 51,52 | szs[a] | | | / |
| Zygomaticomaxillary suture | 53,54 | zms | | | / |
| Interior and superior fissures[b] | 55,56 | isof[a] | | | Manually created two V-shape plates to fill space of the fissures. |
| Lacrimo-ethmoido-maxillary suture[b] | 57,58 | lems[a] | | | / |
| Frontoethmoidal suture - lateral portion | 59,60 | fts(l)[a] | | | / |
| Lateral nasal wall suture | 61,62 | lns[a] | | | / |
| Palatomaxillary and transverse palatine sutures | 63,64 | ptps[a] | | | Combined two sutures. |
| Median palatine suture | 65 | mps | | | / |
| Intermaxillary suture | 66 | ims | | | / |
| Frontoethmoidal suture - upper portion | 67 | fts(u)[a] | | | Segmented as one component which connecting the frontal and ethmoid bones. |
| Frontonasal suture | 68 | fns | | | / |
| Internasal suture | 69 | ins | | | / |
| Nasomaxillary suture | 70,71 | nms | | | / |
| Zygomaticotemporal suture | 72,73 | zts | | | / |
| Intracranial volume | 74 | ICV | Cavity and Volume | Neurocranium | / |
| Orbital volume | 75,76 | OV | | Viscerocranium | / |
| Nasal cavity volume | 77 | NCV | | | / |
| Upper intraoral volume | 78 | UIV | | | / |
| Foramen magnum cover | 79 | FMC[a] | Artificial Cover | Neurocranium | Segmented as one plate which covers the entire foramen magnum. |
| Orbital volume cover | 80 | OVC[a] | | Viscerocranium | Segmented as one plate which covers the entire orbital region through orbital rims. |
| Nasal cavity volume cover | 81 | NCVC[a] | | | Segmented as two plates which cover the entire region of the nasal aperture (first plate), and the posterior nasal choanae (second plate). |
| Upper intraoral volume cover | 82 | UIVC[a] | | | Segmented as one plate which covers the upper intraoral region through the alveolar margin. |

The edges or the contact interfaces of all above components have been flattened, and the width of suture, synchondrosis and fontanelle components has been increased by 2 - 4 mm. Common craniofacial components and anatomical abbreviations are sourced from [S4–S7] unless otherwise noted:
[a] Abbreviations defined by authors.
[b] Component contain more than one sutures or bones.

**Table S2.** Details of 12 cranial measurements, 1 module and 3 indexes used in this study. This table is related to Figure 2 and STAR Methods.

| No. | Measurement | Landmark 1 | Landmark 2 |
|---|---|---|---|
| 1 | Cranial circumference | Glabella (g) | Opisthocranion (op) |
| 2 | Maximum cranial breadth | Euryon (eu) - Right | Euryon (eu) - Left |
| 3 | Maximum cranial length | Glabella (g) | Opisthocranion (op) |
| 4 | Basion-Bregma height | Bregma (b) | Basion (ba) |
| 5 | Upper facial height | Nasion (n) | Prosthion (pr) |
| 6 | Biorbital breadth | Ectoconchion (ec) -Right | Ectoconchion (ec) - Left |
| 7 | Bizygomatic breadth | Zygion (zy) - Right | Zygion (zy) - Left |
| 8 | Nasal breadth | Alare (al) - Right | Alare (al) - Left |
| 9 | Nasal height | Nasion (n) | Nasospinale (ns) |
| 10 | Nasal cavity length | Nasospinale (ns) | Alveolon (alv) |
| 11 | Maximum palatal length | Orale (ol) | Alveolon (alv) |
| 12 | Internal Palatal width (between inter second molars) | Endomolare (enm) - Right | Endomolare (enm) - Left |
| **No.** | **Index** | **Definition** | |
| 1 | Cranial Module (CM) | (Maximum cranial length + Maximum cranial breadth + Basion-Bregma height) / 3 | |
| 2 | Cephalic Index (CI) | Maximum cranial breadth / Maximum cranial length x 100 | |
| 3 | Mid Facial Index (MFI)[a] | Upper facial height / Biorbital breadth x 100 | |
| 4 | Upper Facial Index (UFI) | Upper facial height / Bizygomatic breadth x 100 | |

Definitions of above anatomical landmarks, measurements and indexes are sourced from [S7] unless otherwise noted:
[a] Defined by authors.

**Table S3.** (a) Four cranial volumes (intracranial volume-ICV, orbital volume-OV, nasal cavity volume-NCV and upper intraoral volume-UIV) predicted at each load-step (age) through regression analyses of the normative *in vivo* dataset (n=217). (b) Details of individuals selected as a baseline model at 3 months and as average skulls at 6, 12, 24, 36, and 48 months of age according to cranial volumes. This table is related to Figure 2 and STAR Methods.

**(a)**

| Load-step i | Age (Months) | Intracranial volume (ICV) | Orbit volume (OV) | Nasal cavity volume (NCV) | Upper intraoral volume (UIV) | ICV differences between load-step i and i+1 | OV differences between load-step i and i+1 | NCV differences between load-step i and i+1 | UIV differences between load-step i and i+1 |
|---|---|---|---|---|---|---|---|---|---|
| 0 | 3 | 694.93 | 19.48 | 6.07 | 1.49 | - | - | - | - |
| 1 | 4 | 743.13 | 20.98 | 6.32 | 1.56 | 48.20 | 1.50 | 0.26 | 0.06 |
| 2 | 5 | 788.29 | 22.37 | 6.58 | 1.62 | 45.16 | 1.39 | 0.26 | 0.06 |
| 3 | 6 | 830.51 | 23.65 | 6.84 | 1.69 | 42.23 | 1.28 | 0.26 | 0.06 |
| 4 | 7 | 869.92 | 24.84 | 7.09 | 1.75 | 39.40 | 1.18 | 0.26 | 0.06 |
| 5 | 8 | 906.61 | 25.93 | 7.35 | 1.82 | 36.69 | 1.09 | 0.26 | 0.06 |
| 6 | 9 | 940.70 | 26.93 | 7.60 | 1.88 | 34.09 | 1.00 | 0.26 | 0.06 |
| 7 | 10 | 972.30 | 27.84 | 7.86 | 1.95 | 31.60 | 0.91 | 0.26 | 0.06 |
| 8 | 12 | 1028.46 | 29.42 | 8.37 | 2.08 | 56.16 | 1.58 | 0.51 | 0.13 |
| 9 | 15 | 1096.76 | 31.25 | 9.14 | 2.27 | 68.30 | 1.83 | 0.77 | 0.19 |
| 10 | 18 | 1148.57 | 32.54 | 9.91 | 2.47 | 51.80 | 1.29 | 0.77 | 0.19 |
| 11 | 24 | 1214.54 | 33.96 | 11.44 | 2.86 | 65.97 | 1.42 | 1.54 | 0.39 |
| 12 | 30 | 1250.10 | 34.62 | 12.98 | 3.24 | 35.56 | 0.66 | 1.54 | 0.39 |
| 13 | 36 | 1278.96 | 35.46 | 14.52 | 3.63 | 28.86 | 0.84 | 1.54 | 0.39 |
| 14 | 42 | 1324.85 | 37.43 | 16.05 | 4.02 | 45.89 | 1.97 | 1.54 | 0.39 |
| 15 | 48 | 1411.47 | 41.46 | 17.59 | 4.41 | 86.62 | 4.03 | 1.54 | 0.39 |

*Volume unit: $cm^3$

**(b)**

| Total sample numbers in the relevant range | Selected sample details (Sex_Age in days) | Selected sample age (Months) | ICV | OV) | NCV | UIV | Absolute differences (%) in ICV between selected individual and predicted value | Absolute differences (%) in OV between selected individual and predicted value | Absolute differences (%) in NCV between selected individual and predicted value | Absolute differences (%) in UIV between selected individual and predicted value | Voxel size (mm) |
|---|---|---|---|---|---|---|---|---|---|---|---|
| n=13 (2-4 months) | F_111 | 3.7 | 694.5 | 20.2 | 6.3 | 1.3 | 0% | 4% | 4% | 16% | 0.304*0.304*0.296 |
| n=15 (5-7 months) | M_177 | 5.8 | 889.29 | 23.28 | 6.97 | 1.87 | 7% | 2% | 2% | 11% | 0.400*0.400*0.625 |
| n=13 (11-13 months) | M_348 | 11.4 | 1072.71 | 25.66 | 8.10 | 2.29 | 4% | 13% | 3% | 10% | 0.391*0.391*0.312 |
| n=7 (23-25 months) | F_723 | 23.8 | 1231.26 | 30.48 | 11.89 | 3.00 | 1% | 10% | 4% | 5% | 0.414*0.414*0.312 |
| n=7 (35-37 months) | M_1123 | 36.9 | 1264.54 | 36.06 | 13.59 | 3.16 | 1% | 2% | 6% | 13% | 0.391*0.391*0.625 |
| n=3 (47-48 months) | M_1440 | 47.4 | 1413.09 | 42.02 | 17.85 | 5.75 | 0% | 1% | 1% | 30% | 0.391*0.391*0.312 |

*Volume unit: $cm^3$

**Table S4.** Mesh configurations of the baseline *in silico* model representing 3 months of age. This table is related to Figure 2 and STAR Methods.

| **Component group of baseline *in silico* model** | **Component reference No.** | **Mesh configurations (Applied parameters of element edge length)** | **Mesh optimization** | **Number of elements within component group** | **Volume of component group ($cm^3$)** | **Element edge length (mm)** |
|---|---|---|---|---|---|---|
| Neurocranial bones and Cartilages | 1-10 | Uniform tetrahedral meshing (0.6 mm) | No | 1064023 | 86.19 | Mean: 0.63 Max. - Min.: 0.07 - 1.25 |
| Viscerocranial bones and Cartilages | 11-23 | Uniform tetrahedral meshing (0.6 mm) | No | 265486 | 18.79 | Mean: 0.61 Max. - Min.: 0.09 - 1.45 |
| All sutures, fontanelles and synchondroses | 24-73 | Uniform tetrahedral meshing (0.2 mm) | Increasing element numbers to accommodate minimum tissue differentiation rate (0.2 mm/month, see Table 1 in Main text). | 1971731 | 13.94 | Mean: 0.35 Max. - Min.: 0.07 - 0.81 |
| Intracranial volume (ICV) | 74 | Non-uniform and layered tetrahedral meshing* (Max. 20 mm) | Decreasing element numbers to reduce simulation time. | 1574894 | 694.93 | Mean: 0.79 Max. - Min.: 0.11 - 22.63 |
| Orbital volumes (OV) | 75,76 | Non-uniform and layered tetrahedral meshing* (Max. 6 mm) | | 189152 | 19.48 | Mean: 0.77 Max. - Min.: 0.17 - 6.62 |
| Nasal cavity volume (NCV) | 77 | Non-uniform and layered tetrahedral meshing* (Max. 2 mm) | | 68180 | 6.07 | Mean: 0.73 Max. - Min.: 0.12 - 2.60 |
| Upper intraoral volume (UIV) | 78 | Non-uniform and layered tetrahedral meshing* (Max. 2 mm) | | 17246 | 1.49 | Mean: 0.85 Max. - Min.: 0.19 - 2.20 |
| Artificial Covers | 79-82 | Non-uniform and layered tetrahedral meshing* (0.6 mm) | No | 110602 | 5.99 | Mean: 0.65 Max. - Min.: 0.12 - 1.47 |

*This method increases element edge length with succeeding layers of elements from the edge to center.

**References**

[S1] Libby, J., Marghoub, A., Johnson, D., Khonsari, R.H., Fagan, M.J., and Moazen, M. (2017). Modelling human skull growth: a validated computational model. J. R. Soc. Interface 14, 20170202. https://doi.org/10.1098/rsif.2017.0202.

[S2] Cross, C., Khonsari, R.H., Patermoster, G., Arnaud, E., Larysz, D., Kölby, L., Johnson, D., Ventikos, Y., and Moazen, M. (2022). A computational framework to predict calvarial growth: Optimising management of sagittal craniosynostosis. Front. Bioeng. Biotechnol. 10, 913190. https://doi.org/10.3389/fbioe.2022.913190.

[S3] Marghoub, A., Libby, J., Babbs, C., Pauws, E., Fagan, M.J., and Moazen, M. (2018). Predicting calvarial growth in normal and craniosynostotic mice using a computational approach. J. Anat. 232, 440–448. https://doi.org/10.1111/joa.12764.

[S4] Idriz, S., Patel, J.H., Ameli Renani, S., Allan, R., and Vlahos, I. (2015). CT of normal developmental and variant anatomy of the pediatric skull: Distinguishing trauma from normality. RadioGraphics 35, 1585–1601. https://doi.org/10.1148/rg.2015140177.

[S5] Vu, G.H., Xu, W., Go, B.C., Humphries, L.S., Kalmar, C.L., Taylor, J.A., Bartlett, S.P., Vossough, A., Nah-Cederquist, H.-D., and Swanson, J.W. (2021). Physiologic timeline of cranial-base suture and synchondrosis closure. Plast. Reconst. Surg. 148, 973e. https://doi.org/10.1097/PRS.0000000000008570.

[S6] Wang, M.M., Haveles, C.S., Zukotynski, B.K., Reid, R.R., and Lee, J.C. (2022). The 27 facial sutures: Timing and clinical consequences of closure. Plast. Reconst. Surg. 149, 701–720. https://doi.org/10.1097/PRS.0000000000008816.

[S7] Liang, C., Profico, A., Buzi, C., Khonsari, R.H., Johnson, D., O’Higgins, P., and Moazen, M. (2023). Normal human craniofacial growth and development from 0 to 4 years. Sci. Rep. 13, 9641. https://doi.org/10.1038/s41598-023-36646-8.
